# Metabolic Phenotyping of Marine Heterotrophs on Refactored Media Reveals Diverse Metabolic Adaptations and Lifestyle Strategies

**DOI:** 10.1128/msystems.00070-22

**Published:** 2022-07-20

**Authors:** Elena Forchielli, Daniel Sher, Daniel Segrè

**Affiliations:** a Department of Biology, Boston University, Boston, Massachusetts, USA; b Biological Design Center, Boston University, Boston, Massachusetts, USA; c Department of Marine Biology, Leon H. Charney School of Marine Sciences, University of Haifa, Haifa, Israel; d Bioinformatics Program, Boston University, Boston, Massachusetts, USA; e Department of Physics and Department of Biomedical Engineering, Boston University, Boston, Massachusetts, USA; University of Massachusetts Medical School

**Keywords:** carbon sources, heterotrophic bacteria, marine microbiome, metabolism, microbial diversity, microbial ecology, phenotyping, systems biology

## Abstract

Microbial communities, through their metabolism, drive carbon cycling in marine environments. These complex communities are composed of many different microorganisms including heterotrophic bacteria, each with its own nutritional needs and metabolic capabilities. Yet, models of ecosystem processes typically treat heterotrophic bacteria as a “black box,” which does not resolve metabolic heterogeneity nor address ecologically important processes such as the successive modification of different types of organic matter. Here we directly address the heterogeneity of metabolism by characterizing the carbon source utilization preferences of 63 heterotrophic bacteria representative of several major marine clades. By systematically growing these bacteria on 10 media containing specific subsets of carbon sources found in marine biomass, we obtained a phenotypic fingerprint that we used to explore the relationship between metabolic preferences and phylogenetic or genomic features. At the class level, these bacteria display broadly conserved patterns of preference for different carbon sources. Despite these broad taxonomic trends, growth profiles correlate poorly with phylogenetic distance or genome-wide gene content. However, metabolic preferences are strongly predicted by a handful of key enzymes that preferentially belong to a few enriched metabolic pathways, such as those involved in glyoxylate metabolism and biofilm formation. We find that enriched pathways point to enzymes directly involved in the metabolism of the corresponding carbon source and suggest potential associations between metabolic preferences and other ecologically relevant traits. The availability of systematic phenotypes across multiple synthetic media constitutes a valuable resource for future quantitative modeling efforts and systematic studies of interspecies interactions.

**IMPORTANCE** Half of the Earth’s annual primary production is carried out by phytoplankton in the surface ocean. However, this metabolic activity is heavily impacted by heterotrophic bacteria, which dominate the transformation of organic matter released from phytoplankton. Here, we characterize the diversity of metabolic preferences across many representative heterotrophs by systematically growing them on different fractions of dissolved organic carbon. Our analysis suggests that different clades of bacteria have substantially distinct preferences for specific carbon sources, in a way that cannot be simply mapped onto phylogeny. These preferences are associated with the presence of specific genes and pathways, reflecting an association between metabolic capabilities and ecological lifestyles. In addition to helping understand the importance of heterotrophs under different conditions, the phenotypic fingerprint we obtained can help build higher resolution quantitative models of global microbial activity and biogeochemical cycles in the oceans.

## INTRODUCTION

Three quarters of Earth’s surface is covered in water, making the ocean the biggest continuous environment and home to extraordinary biodiversity (1). In stark contrast to terrestrial biomes, approximately 70 percent of the biomass in marine ecosystems is microbial (versus 96% plant on land) (2); accordingly, their submicroscale processes have global-scale consequences on ecosystem services critical to human society (3). Half of the Earth’s annual primary production is accomplished in the surface ocean by phytoplankton that harvest light to fix carbon dioxide (3–5). Heterotrophic bacteria are important players in these processes, as they impact carbon cycling in the marine environment along at least two main axes: first, phytoplankton function is modulated by interactions with heterotrophs in ways that drastically affect primary productivity (6–9). For example, heterotrophic bacteria provide nutrients essential for the long-term survival of some phytoplankton (10–13). Second, heterotrophs dominate the transformation of organic matter released from phytoplankton, and their metabolic activity ultimately determines the fate of organic carbon in the marine environment (6, 14).

The marine dissolved organic carbon (DOC) pool is the primary source of organic carbon for marine heterotrophs, containing an estimated 0.2 Pg of labile organic compounds, which can be readily metabolized by these bacteria (15). Analyses of bulk seawater reveal the enormous complexity and heterogeneity of marine DOC, enumerating a minimum of tens of thousands of distinct organic compounds (16–18). As the primary suppliers of marine DOC, phytoplankton transfer approximately 50% of their photosynthate to heterotrophs (4, 19) via a variety of active and passive mechanisms, including leakage (20), exudation (21), photosynthetic overflow (22), and cell death caused by viral lysis and protist grazing (23, 24). A number of studies indicate that the taxonomic composition of microbial communities is influenced by the provenance of organic material, suggesting that individual heterotrophs vary in their ability to utilize broadly defined classes of macromolecules (25, 26) and that these metabolic preferences may contribute to community structure (27–31). For example, heterotrophs associated with phytoplankton have shown preferences for amino acids, small sulfur-containing compounds, and one-carbon compounds (32–36). However, little is known about the specific preferences of individual heterotrophs for individual classes of compounds, making it difficult to understand the role that specific clades play in utilizing DOC in different environments. This limited knowledge also makes it challenging to lay the groundwork for mechanistic models that could help explain how such interactions shape the phylogenetic and functional composition of the community. Identifying the metabolic links between DOC and heterotrophs is key to understanding the ecological drivers underpinning carbon cycling in ocean ecosystems. This may also help improve global-scale models of marine microbial processes, where heterotrophs are often assumed to perform overall similar metabolic tasks (37, 38), an assumption likely reasonable for certain goals (e.g., modeling global primary productivity) but not others (e.g., modeling community composition or genetic capacity).

In principle, information on the substrate preferences of different heterotrophs could be inferred from their genomes; however, sequencing data alone have demonstrated a limited ability to predict microbial phenotypes and community functions in practice (39–43). For example, in a recent survey of human gut bacteria, metabolic models recapitulated growth for only 10 of the 40 strains tested, suggesting that genomic information combined with knowledge from the literature is insufficient to describe bacterial metabolic complexity (44). Alternatively, by measuring the growth properties of individual strains on specific carbon sources, one can infer phenotypic profiles that provide direct insight into metabolic preferences and growth strategies. These types of measurements are increasingly performed to characterize microbial collections from different biomes (45, 46). Ideally, one would want to analyze phenotypic profiles in conjunctions with the organisms’ genomes in order to obtain insight into the genes and pathways that confer these preferences. Performing these types of measurements on well-defined synthetic media, in addition to enabling inferences regarding the specific metabolic capacities of microorganisms, could also help inform quantitative models (47, 48).

Here we report the generation and characterization of a collection of 63 heterotrophic bacteria representative of many major marine clades and a systematic analysis of their metabolic preferences. By designing simplified media that capture different fractions of the molecular components of marine DOC, we sought to characterize the metabolic properties of different representative heterotrophs, i.e., how they grow on different conditions that represent different axes of DOC. We next analyzed the phenotypes obtained in order to understand whether these metabolic phenotypes are well captured by phylogeny or other genome-encoded properties. We suggest that our analysis could contribute to helping reduce the complexity of the ocean microbiome to a set of computationally and experimentally tractable variables that can be interrogated with mathematical models and controlled experiments and extended to complex natural communities to explain aspects of their behavior.

## RESULTS AND DISCUSSION

### Growth of 63 heterotrophs on refactored media provides an atlas of their metabolic preferences.

In order to generate a collection of strains representative of major marine lineages common to both the global oligotrophic and temperate oceans, we collected 63 heterotrophic isolates from different sources (Table S1 in supplemental material). Strains were selected via a comprehensive genomic analysis conducted in a recent effort (49), in which over 400 high-quality reference genomes were clustered into functional groups using a trait-based approach focusing on metabolism and microbial interactions. Representative strains were chosen from each functional cluster, applying the additional criteria that they be culturable in standard laboratory conditions (at 26°C in Marine Broth) and have a biosafety level 1 rating. In addition, we also included some nonmarine model strains to serve as a benchmark for our experiments (see Materials and Methods). Overall, the culture collection includes representatives of 5 phyla and 29 families.

10.1128/msystems.00070-22.7 TABLE S1 Nicknames, taxonomy, and sources of all strains in the library. Download Table S1, XLSX file, 0.01 MB. Copyright © 2022 Forchielli et al. 2022 Forchielli et al. https://creativecommons.org/licenses/by/4.0/ This content is distributed under the terms of the Creative Commons Attribution 4.0 International license.

**FIG 1:**
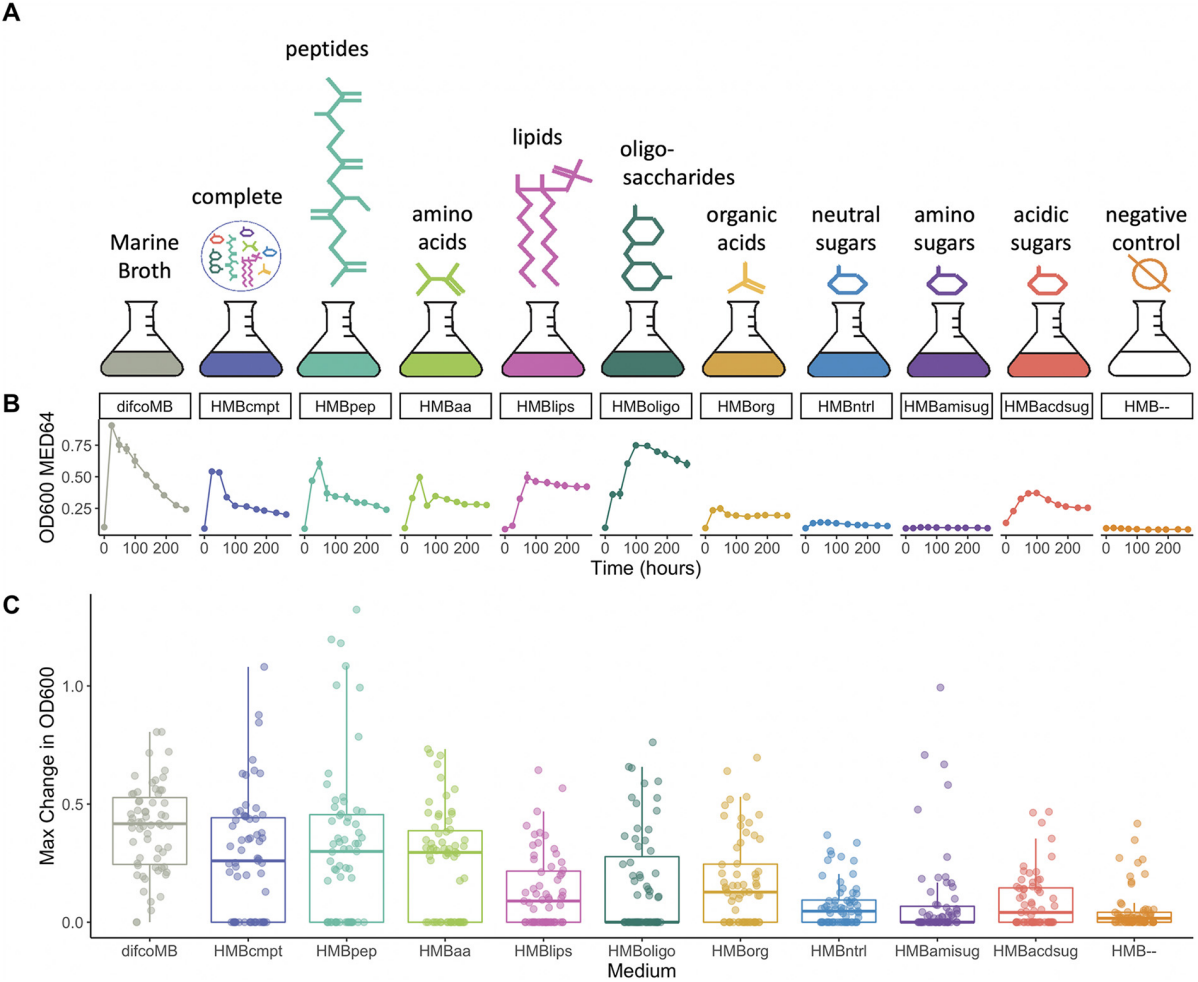
An overview of the experimental setup. Each strain in the heterotroph library was grown individually on each of the 11 different media. (A) Difco Marine Broth was refactored into 8 carbon classes, which were used to make defined media. The set of media used in the experiment also includes a medium containing all carbon categories (“complete”) and a negative control lacking added carbon. (B) Growth was assayed by measuring the OD_600_ over the course of 264 h; *Alteromonas mediterranea* MED64 growth curves are shown here as an example. (C) MaxOD, defined as the maximum OD_600_ reached during the growth process (relative to the culture at time zero), was determined for each strain under every possible medium. Here, for each medium (*x* axis) we visualize the MaxOD of each organism as a dot (with small random displacement on the *x* axis for ease of visualization). The box represents the median and quartiles of the MaxOD values. Note: some organisms display small but reproducible nonzero growth on HMB–, possibly due to an ability to utilize other media components or internal carbon storage molecules for growth.

We used this collection of heterotrophic bacteria to address, through single strain phenotyping, the question of whether different clades are geared toward efficient degradation or preferred utilization of specific subsets of DOC molecules. Many of the library strains had been reported to grow in undefined complex media, such as Marine Broth. Using the composition of Marine Broth as a scaffold, we refactored yeast extract and peptone (the primary carbon sources in Marine Broth) into eight types of organic carbon (hereafter referred to simply as “carbon classes”): peptides, amino acids, lipids, disaccharides, organic acids, neutral sugars, amino sugars, and acidic sugars (Fig. 1A). For some classes, we selected specific compounds based on their reported presence in marine DOC (50–52). For example, in the amino sugar class, *N*-acetylglucosamine was chosen because it is a known degradation product of chitin, the primary cell wall component of many marine organisms (53). All refactored media except HMBlips were calibrated to contain the same total mass of carbon-containing compounds (although not necessarily the same number of carbon atoms, see Fig. S4 and Supplementary Text S1), as well as an excess of nitrogen, phosphorus, sulfur, salts, trace metals, and vitamins (see Materials and Methods and Table S2). Together with a negative control lacking added organic carbon, a medium containing all carbon classes, and Marine Broth itself, we tested a total of 11 conditions using growth assays in 96-well plates (Fig. 1B).

10.1128/msystems.00070-22.4 FIG S4 Bacterial growth patterns explained by media stoichiometry. While media were built to have equivalent total amounts of carbon sources, they differ in their elemental stoichiometry, potentially affecting growth patterns. We performed linear regression to test the hypothesis that the quantity of growth can be attributed to the number of carbon atoms (A), nitrogen atoms (C), or the number of compounds (E) in a medium. There is a significant but weak linear relationship between the number of carbon (A) and nitrogen (C) atoms and change in OD_600_ (adjusted *R*^2^ = 0.022 and *P* = 9.5 × 10^−5^ for C; adjusted *R*^2^ = 0.079 and *P* = 3.5 × 10^−13^ for N). Regression analysis for individual strains: 4 and 22 out of 63 strains display a statistically significant relationship between growth and moles of carbon (B) and nitrogen (D), respectively (Table S9 at [https://github.com/segrelab/marine_heterotrophs/]). The average change in OD_600_ per added mol was much greater for nitrogen than carbon: 6.97 and 0.586 OD_600_/mol for N and C, respectively (Table S9 at https://github.com/segrelab/marine_heterotrophs/). We performed linear regression to examine whether bacterial growth could be explained by the number of carbon sources added to the media (E), and found a significant but weak linear relationship (adjusted *R*^2^ = 0.041 and *P* = 1.61 × 10^−7^). Examined individually, 6 of the 63 strains displayed significant relationships between the number of compounds and growth (F), but the effect is small: across all strains, the average change in OD_600_ per additional compound is only 0.0053 (Table S9 at https://github.com/segrelab/marine_heterotrophs/). In all panels, each dot represents the maximum average change in OD_600_ for a single strain. DifcoMB is not pictured in Fig. S4 because the amount of added carbon, added nitrogen, and number of compounds cannot be determined. Download FIG S4, TIF file, 2.2 MB. Copyright © 2022 Forchielli et al. 2022 Forchielli et al. https://creativecommons.org/licenses/by/4.0/ This content is distributed under the terms of the Creative Commons Attribution 4.0 International license.

10.1128/msystems.00070-22.8 TABLE S2 Recipes for the defined media. Download Table S2, XLSX file, 0.02 MB. Copyright © 2022 Forchielli et al. 2022 Forchielli et al. https://creativecommons.org/licenses/by/4.0/ This content is distributed under the terms of the Creative Commons Attribution 4.0 International license.

10.1128/msystems.00070-22.9 TEXT S1 Impact of media stoichiometry on growth. Download Text S1, DOCX file, 0.01 MB. Copyright © 2022 Forchielli et al. 2022 Forchielli et al. https://creativecommons.org/licenses/by/4.0/ This content is distributed under the terms of the Creative Commons Attribution 4.0 International license.

**FIG 2:**
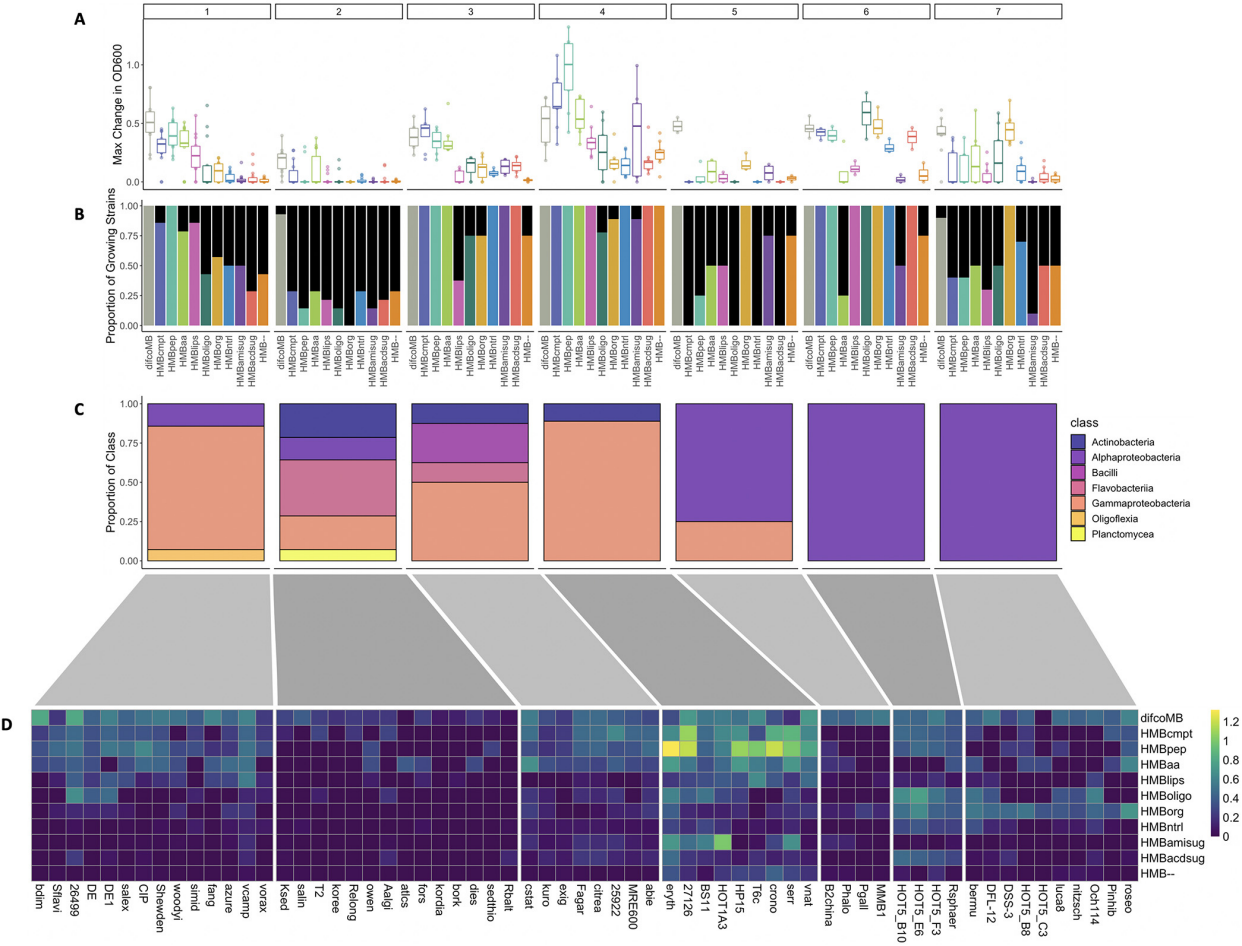
Clustering of growth profiles (defined as the vector of MaxOD values for each organism across all media) reveals metabolic signatures of the heterotroph library. (A) A representation of MaxOD for each cluster. Each subplot (numbered from 1 to 7) shows a boxplot of the MaxOD across media (similar to the one of Fig. 1C), but restricted to the organisms belonging to the corresponding cluster. For example, cluster 4 includes organisms which grow very well on the peptide-containing medium (HMBpep). (B) The proportion of strains determined to have positive growth (see Materials and Methods) on each medium, for each of the clusters. For example, in cluster 4, all species grow on the first four media. (C) The proportion of taxonomic groups represented in each cluster (at the class level, see color legend to the right). (D) Clustered heatmap depicting the MaxOD for each strain/medium combination. For each cluster, the heatmap (media, *y* axis by organism, *x* axis) shows the MaxOD value (see color code to the right of the panel). 5 of the 63 strains are considered nonmarine in origin: 25922, MRE600, crono, shewden, and exig.

All of the 63 marine heterotroph strains grew reproducibly on at least one of the defined media, and optical density (OD) measurements were almost identical across technical replicates and highly consistent across biological replicates (Fig. 2; Fig. S1). For subsequent analyses we focused on the maximal OD (maxOD) observed along the curve of each strain relative to time zero, which we took as a proxy for the efficiency with which each organism can produce biomass on a given carbon category (referred to henceforth also as “productivity”). Note that another important metric, the growth rate at log phase is strongly correlated with maxOD (adjusted *R*^2^ = 0.71, *P* < 2.2 × 10^−16^; Fig. S2). Despite containing the same mass of carbon source and supporting roughly similar numbers of strains (Fig. S3), the different media led to very different degrees of productivity. Given that neutral sugars, such as glucose and arabinose, are classically used as preferred carbon sources in bacterial growth experiments, we were surprised to observe that media having peptides and amino acids as the main carbon sources supported the highest amount of biomass growth, while the neutral sugar medium was among the lowest supporters of biomass change (Fig. 1C).

10.1128/msystems.00070-22.1 FIG S1 Analysis of experimental reproducibility for the growth measurements, through the comparison of biological replicates. (A) Each panel displays all growth measurements across the different media for a given strain. The 19 panels correspond to the 19 strains for which 4 different biological replicates (each with 3 technical replicates) were assessed. For other strains, the measurements involved 2 or 3 biological replicates (see GitHub repository for corresponding dataset). In each panel, the box plots reflect the distributions of all the (4 × 3) different repeats. Spread of these repeats varies across strains and conditions, and is greater for larger OD values. (B) All MaxOD measurements for different strains on different media are compared across different pairs of biological replicate experiments. Each dot represents the average of three technical replicates. (C) The distribution of standard deviation values between technical replicates for all combinations of strains and experiments. Overall, the median of this distribution of standard deviations (~0.01, dashed red line) is much lower than the average change in OD reached across all experiments (~0.1) supporting the high reproducibility of the experimental setup. Download FIG S1, TIF file, 1.0 MB. Copyright © 2022 Forchielli et al. 2022 Forchielli et al. https://creativecommons.org/licenses/by/4.0/ This content is distributed under the terms of the Creative Commons Attribution 4.0 International license.

10.1128/msystems.00070-22.2 FIG S2 The scatter plot displays the maximum change in OD_600_ (MaxOD, see Methods) as a function of the maximum growth rate (OD_600_/hour) for all strain/medium combinations. The maximum growth rate was identified for each growth curve as the maximal slope observed across neighboring time points. There is a strong positive correlation between the two (adjusted *R*^2^ = 0.68, *P* < 2.2 × 10^−16^). Download FIG S2, TIF file, 0.4 MB. Copyright © 2022 Forchielli et al. 2022 Forchielli et al. https://creativecommons.org/licenses/by/4.0/ This content is distributed under the terms of the Creative Commons Attribution 4.0 International license.

10.1128/msystems.00070-22.3 FIG S3 The number of strains that display positive growth on each medium. Growth was considered positive if statistically significantly greater than the negative control sample lacking added bacteria (see methods, Table S7 at https://github.com/segrelab/marine_heterotrophs/). Download FIG S3, TIF file, 0.3 MB. Copyright © 2022 Forchielli et al. 2022 Forchielli et al. https://creativecommons.org/licenses/by/4.0/ This content is distributed under the terms of the Creative Commons Attribution 4.0 International license.

One aspect of the above results that is worth reflecting on is the significance of this pattern in the context of prior observations. It has been suggested in the literature that marine heterotrophic bacteria achieve greater biomass yield and growth rates on amino acids compared to media containing mono or polysaccharides as the sole carbon source (54, 55). The reason for this is unclear, but field studies suggest that nitrogen limitation relieved by the presence of additional nitrogen in amino acids does not explain this discrepancy (55). We did not observe increased growth on the amino sugar-containing medium (among the lowest growing), which contains considerably more nitrogen than the nonamino media. Furthermore, a regression analysis showed that there is only a weak relationship between the change in OD and amount of nitrogen in the medium (adjusted *R*^2^ = 0.079 and *P* = 3.5 × 10^−13^; Fig. S4C). Thus, nitrogen abundance is likely not the main or only reason for the extensive bacterial growth on amino acids, and other explanations could involve the energetic advantage of using preformed amino acids compared to their biosynthesis (56). For a complete discussion on the influence of media stoichiometry on growth, see Supplementary Text S1 and Fig. S4. Note that some strains reproducibly displayed nonnegligible growth on the medium with no carbon added (see Table S7 at https://github.com/segrelab/marine_heterotrophs/ for *P* values). The high reproducibility between experiments (Fig. S1) likely rules out contamination. Carryover of nutrients from the starter culture is also unlikely to explain this, since these strains grew to a lesser magnitude on other media (unless inhibition by carbon in those other media is a contributing factor). We cannot exclude the possibility that these strains are capable of utilizing compounds other than the added carbon sources (e.g., vitamins [57]) or internal carbon stores for growth (58, 59) or that they possess autotrophic capabilities (60).

### Organisms cluster into two main groups and finer structures based on metabolic preferences.

We captured each strain’s metabolic phenotype by compiling a growth profile, which consisted of a vector of the maximum change in OD achieved by the strain on each medium. Using a Gaussian mixture model (see Materials and Methods), these 63 growth profiles could be divided into 7 clearly distinct clusters (Fig. 2) The clusters can be described in terms of unique metabolic “signatures,” i.e., distinct sets of carbon classes on which the strains achieved similar biomass growth.

At a very broad level, six clusters seem to partition into two categories of metabolic preferences: one group (clusters 1, 3, and 4) includes organisms that grow robustly on amino acids and relatively poorly on organic acids (Fig. 2A and B); these clusters are enriched in *Gammaproteobacteria* (squared standardized Pearson residual for chi-square test > 4; see methods, Table S3 at https://github.com/segrelab/marine_heterotrophs/). In contrast, clusters 6 and 7 are comprised of organisms that produce significantly more biomass when grown on the organic acid medium compared to the amino acid medium (Fig. 2A and B; Table S4 at https://github.com/segrelab/marine_heterotrophs/); these clusters are strongly enriched in *Alphaproteobacteria* (squared standardized Pearson residual for chi-square test > 4; see methods, Table S3 at https://github.com/segrelab/marine_heterotrophs/). Cluster 5 follows a similar pattern to clusters 6 and 7, but the magnitude of growth is lower and the observed differences are not statistically significant (Fig. 2A and B). The remaining cluster (2) is highly diverse phylogenetically, albeit strongly enriched for *Flavobacteriia* and *Actinobacteria* (squared standardized Pearson residual for chi-square test > 4; see methods, Table S3 at https://github.com/segrelab/marine_heterotrophs/). As a whole, the strains in cluster 2 do not grow robustly on any media type, which may indicate a requirement for growth factors or environmental conditions not represented in the media tested.

In addition to these broad-scale patterns, the strains in several of the clusters show more nuanced differences in their growth phenotypes. Firstly, although the strains in cluster 4 are capable of growing on organic acids, the magnitude of growth is greatly reduced compared to the media containing amino acids (Fig. 2A; Table S4 at https://github.com/segrelab/marine_heterotrophs/). These strains consistently grow to a higher OD on the negative control medium compared to the organic acids, although this difference is not statistically significant (Fig. 2A; Table S4 at https://github.com/segrelab/marine_heterotrophs/); this might suggest that organic acids actually inhibit their growth. This possibility seems apparently inconsistent with the fact that these strains grow well on HMBcmpt, which also contains organic acids. However, the concentration of organic acids in HMBorg is approximately 47 mM, 8 times higher than the concentration of organic acids in the HMBcmpt. Organic acids are well-known by-products of aerobic growth in some bacteria, and several studies indicate that they inhibit growth in a concentration-dependent manner (61–63). The concentration of acetate alone in HMBorg is 8.7 mM, and in Escherichia coli, acetate concentrations as low as 8 mM have been shown to reduce growth by 50% (64); therefore, our results are consistent with a potential concentration-dependent inhibitory effect by organic acids. In contrast, the strongest growth on organic acids is observed for clusters 6 and 7, which also display a limited and varied ability to grow on amino acids. Four of the 18 strains in clusters 5, 6, and 7 grow appreciably on both peptides and amino acids (Fig. 2B); the remaining 14 either grow on one or neither of these two media. Since growth on peptides requires the ability to take up and incorporate amino acids, it is unlikely that these strains lack this capability. Rather, it is possible that some of the amino acids negatively affect growth at the concentrations employed here (65). Overall, except for cluster 2, which seems to include strains with a common pattern of low OD but no specific preference for carbon classes, the heterotrophs’ metabolic functions are thus primed for optimized growth on amino acids or organic acids, but not both.

### Phylogenetic and metabolic distances correlate poorly with metabolic preference distances.

**FIG 3:**
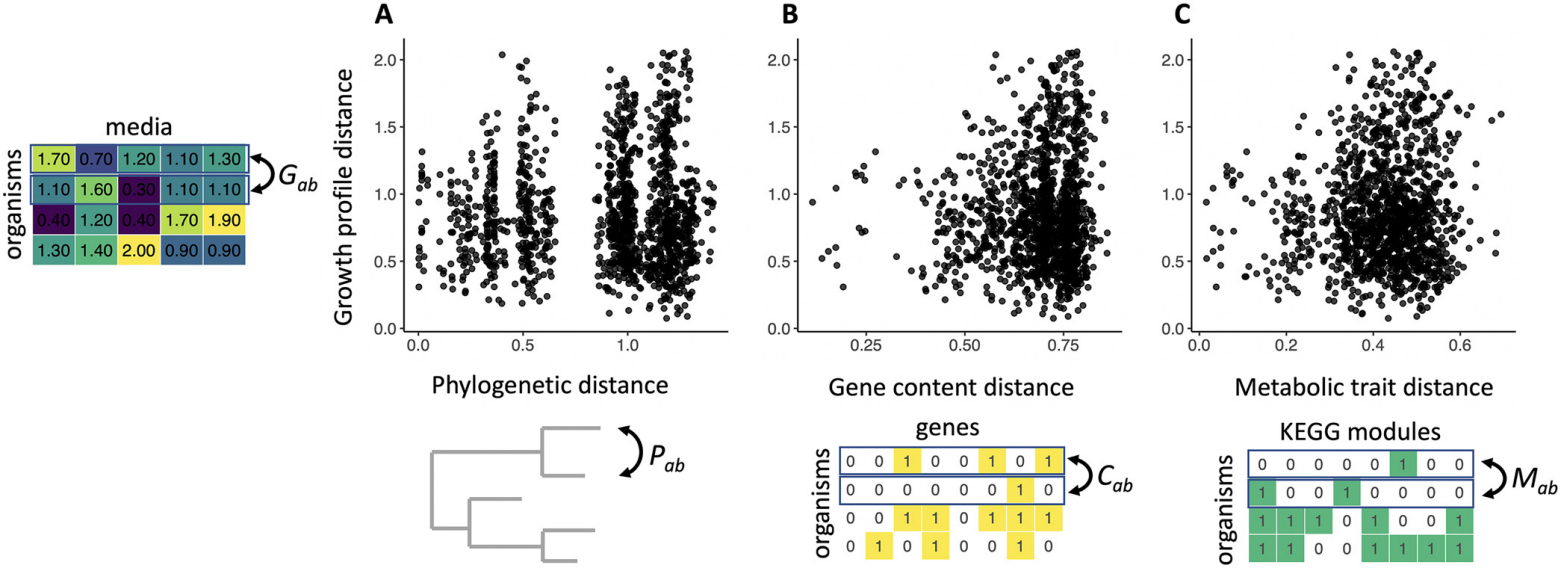
Genome-based distances between all pairs (*a*,*b*) of strains. Phylogenetic distance *P_ab_* (A), genome-wide gene content distances *C_ab_* (B), and KEGG module distance *M_ab_* (C) (see Materials and Methods) are compared to the phenotypic distance *G_ab_* between all pairs of growth profiles shown in Fig. 2D. All of the genome-based distances are poor predictors of the distance between the corresponding growth profiles. The *y* axes in all panels correspond to the Euclidean distance between growth profiles of continuous maxOD values; the *x* axes correspond to the cophenetic distance (A) and Jaccard distance between binary vectors (B and C).

We subsequently asked whether strains that are more closely related to each other phylogenetically are also more similar in their growth profiles across the different media we tested (see Materials and Methods). Regression analysis found an extremely weak relationship between the two variables (Fig. 3A; adjusted *R*^2^ = 0.001, *P* = 0.04), indicating that the differences in phylogenetic distances between strains explained an insignificant proportion of the variation in distance between growth profiles.

Subsequently, we asked whether a metric other than phylogenetic distance, e.g., the difference in gene content, would correlate more strongly with phenotypic distance. Specifically, we compared distances between growth profiles to distances between genomes represented as binary sequences of genes (see Materials and Methods). Linear regression revealed an inconsequential association between growth profile distance and genome distance (Fig. 3B; adjusted *R*^2^ = 0.004, *P* = 0.0005). The same was true when we compared the growth profile distance to the distance between Kyoto Encyclopedia of Genes and Genomes (KEGG) modules, which may better approximate the metabolic distance between strains (Fig. 3C; adjusted *R*^2^ = 0.004, *P* = 0.0004) (49). Taken together, global genomic data, at least with the metrics used so far, do not seem to capture the differences in metabolic phenotypes observed between strains. One possible explanation for our observations is that the genes key in differentiating growth on various carbon sources are “drowned out” by the noise of uninformative genes contained in the genomes; it is not clear that the genes that matter rise above genes that have no effect in this analysis.

### Genes and pathways associated with growth on specific media reflect different metabolic and ecological strategies.

**FIG 4:**
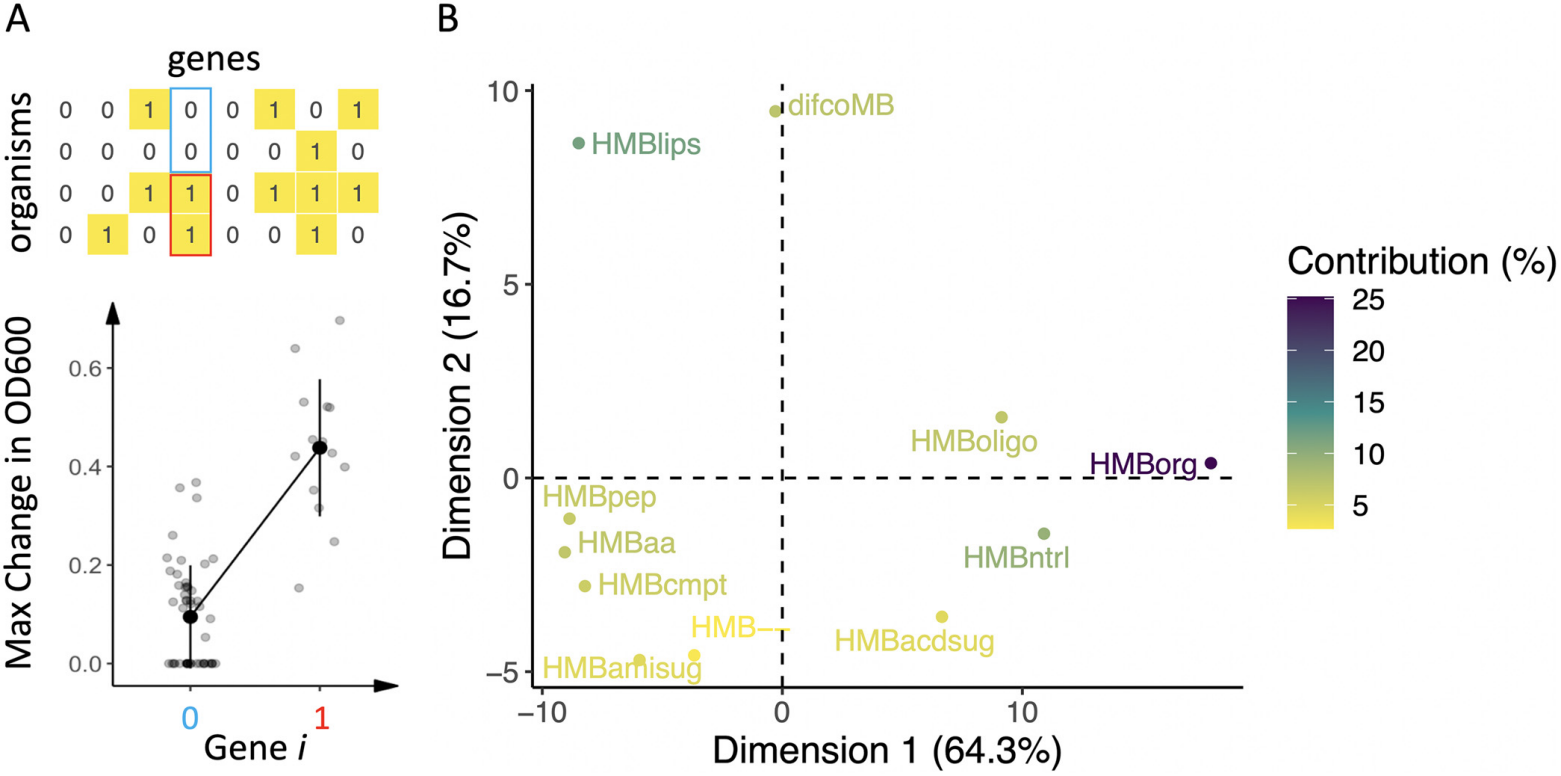
(A) A schematic representation of the process for generating gene-specific correlations between presence/absence of each gene and growth on a given medium. For each medium and each gene, we plot MaxOD versus gene presence/absence for all organisms, and compute the point biserial correlation (see Materials and Methods). This gives rise to a matrix of correlations indicating how much the presence of each gene is predictive of growth (across all organisms) on a given condition. This matrix can also be viewed as a collection of row vectors (of length equal to the number of genes), each representing a condition. (B) Through dimensionality reduction of these row vectors (with PCA) we visualize how similar the media are to each other, in terms of the genes correlated with growth. The first two PCA axes account for 81% of the variance. Data points are colored according to the contribution of the samples (media) to the principal components.

Given the poor correlation between metabolic distance and gene content or phylogenetic distance, we asked whether a more informative relationship could be revealed by examining whether the presence of individual genes or sets of genes (pathways) is associated with growth on each medium. For each medium, we calculated the correlation (r) between growth and each gene in the library pangenome (see Materials and Methods and Fig. 4A). Looking at the list of individual significantly correlated genes (Table S5 at https://github.com/segrelab/marine_heterotrophs/), one clear pattern that emerges is that 386 of the 1,076 most strongly correlated genes (absolute value of *r* > 0.5) are associated with growth on the organic acids medium. Conversely, no significant individual genes emerged for growth on the amino acid medium, despite the fact that this medium supports high growth in a number of strains. One possible interpretation of this difference is that while organisms growing on organic acids tend to use a narrow and fairly coherent set of pathways, the organisms that grow on the amino acid medium are utilizing different subsets of the 20 amino acids, and thus different biological pathways (see Table S2 for details). A concise view of all gene correlation scores can be visualized using principal component PCA: as shown in Fig. 4B, the organic acid medium stands out as the main contributor to the primary PCA axis and is thus the most unique in terms of enriched genes compared to the other media. It is also interesting that the media containing additional nitrogen in the form of amino groups appear to cluster together, suggesting that genes related to the utilization of organic nitrogen partially drive this clustering.

In order to better identify genomic signatures associated with growth phenotypes beyond individual genes, we implemented a gene set enrichment analysis (GSEA) to identify overrepresented pathways. We mapped the set of highly correlated genes to the KEGG database (see Materials and Methods), which resulted in a ranked list of pathways for each medium (Table S6 at https://github.com/segrelab/marine_heterotrophs/); we will refer to these pathways simply as condition-specific “enriched pathways.” We next asked whether the enriched pathways are indicative of the medium under which the enrichment was identified. In other words, are the growth media enriched for pathways that metabolize the class of carbon substrate they contain?

**FIG 5:**
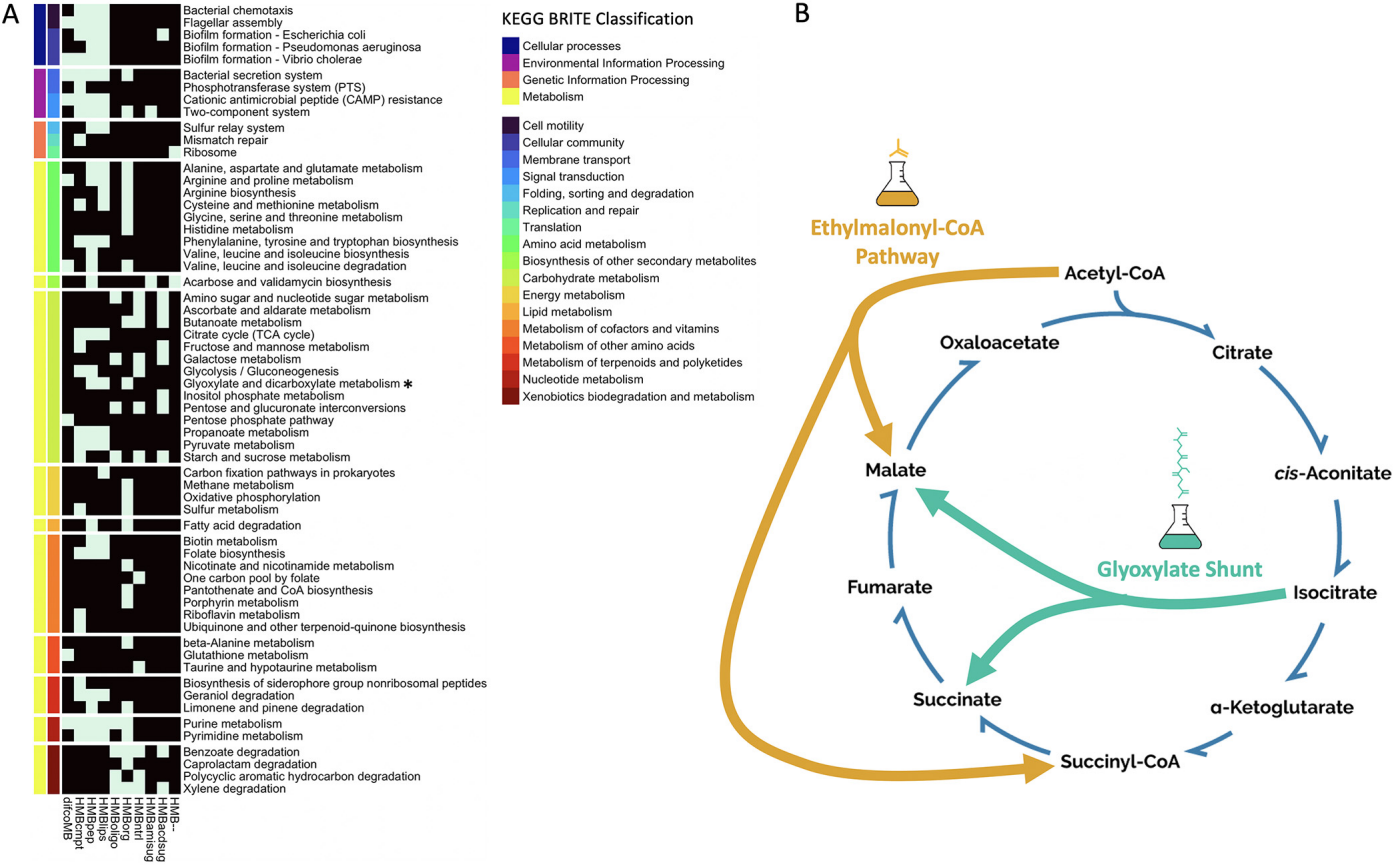
Growth on each medium is associated with specific biological pathways, as determined by a gene set enrichment analysis (see Materials and Methods). (A) The set of highly correlated genes for each medium was mapped to the KEGG pathway database; for each medium, pathways that are significantly overrepresented in the highly correlated genes are colored light blue (black indicates a lack of statistical enrichment). KEGG pathways are annotated with colors corresponding to their BRITE database classification. (B) One of the interesting pathways that emerges from this analysis is the ethylmalonyl CoA pathway (EMC) pathway, which appears in KEGG as part of the Glyoxylate and Dicarboxylate Metabolism pathway (asterisk in panel A). The EMC pathway is strongly enriched for growth on organic acids. This pathway is a multifunctional pathway, known to be usable for organic acid assimilation as an alternative to the glyoxylate shunt.

In several cases, growth on the various media was associated with the KEGG pathways describing the metabolism of the specific compounds they contained. For example, the sugar-based media (HMBoligo, HMBntrl, HMBacdsug) are depleted of pathways involved in the metabolism of amino acids and enriched for pathways involved in galactose, starch and sucrose metabolism, and interconversions between the pentose monosaccharides and glucuronate, a degradation product of alginate (Fig. 5A) (52). The media lacking sugars are not enriched for these sugar degradation pathways; instead, they share an association with the glyoxylate and dicarboxylate metabolism pathway, which can replenish sugars from amino acid precursors. In other cases, growth on a specific category of carbons is associated with pathways that can be involved in the utilization of those compounds but deviate from the most basic expectation. For example, organisms growing on organic acids are enriched for specific portions of the ethylmalonyl-CoA pathway (EMC) (Fig. 5A; Fig. S5A), a well-described method for the assimilation of two-carbon compounds and biosynthesis of carbohydrates from fatty acids (66). Notably, the EMC pathway is an alternative to the glyoxylate shunt (67, 68), which is used to feed anapleurotic reactions of the TCA during growth on C2 substrates, such as acetate (Fig. 5B). A key enzyme in glyoxylate pathway, isocitrate lyase, is known to be absent in certain marine bacteria, such as Rhodobacter sphaeroides, which nonetheless possess the ability to grow on acetate as a sole carbon source (69).

10.1128/msystems.00070-22.5 FIG S5 KEGG pathway enrichment for growth on organic acids. Genes highly correlated with growth on organic acids are mapped onto the Glyoxylate and Dicarboxylate (A) and the Porphyrin and Chlorophyll (B) Metabolism KEGG pathways; the colors represent the sign and strength of the correlation, ranging from green (–1) to red (1). In panel A, the portions of the KEGG pathway representing the Ethylmalonyl-CoA pathway are denoted by the dashed gold boxes. Download FIG S5, TIF file, 2.5 MB. Copyright © 2022 Forchielli et al. 2022 Forchielli et al. https://creativecommons.org/licenses/by/4.0/ This content is distributed under the terms of the Creative Commons Attribution 4.0 International license.

Not all pathways found to be enriched can be easily associated with degradation of the carbon sources included in the medium: these pathways seem to have no direct relationship to the molecular processes associated with the utilization of the corresponding media components. For example, growth on organic acids is also correlated with the KEGG pathway for porphyrin metabolism, specifically the complete pathways for vitamin B12/cobalamin biosynthesis as well as bacteriochlorophyll a and b (Fig. S5B). On the other hand, the folate biosynthesis pathway is enriched for growth on peptides, lipids, and the complete medium (Fig. 5A). In addition, growth on peptides (and lipids) is associated with pathways for motility, including chemotaxis and flagellar assembly, as well as for biofilm formation (Fig. 5A).

### Conclusions.

By defining a suite of carbon compound classes that together make up biomass and asking which marine bacteria grow on each class, we generated a map of the broad-scale metabolic preferences of these bacteria. This map is important because heterotrophs process much of the dissolved oceanic carbon, in ways that may depend on the emergent metabolic properties of microbial communities to which they belong. Obtaining a clear picture of the carbon utilization capabilities of individual strains in relationship to their taxonomic signature seems, therefore, an essential step toward understanding how heterotrophic bacteria impact carbon cycling in the marine environment. Understanding individual strain traits will further allow researchers to know if it is feasible and reasonable to use a few representative taxa and metabolic processes as a general effective description of heterotrophic metabolism in the oceans, e.g., for the purpose of implementing mechanistic models of communities and global ocean biogeochemical cycles. At the same time, irrespective of the details of the phylogeny-metabolism relationship, one can view the broad-scale approach presented here as a valuable effort toward building more informed dynamical models of ecological processes. In particular, we suggest that partitioning heterotrophs into clusters based on their preferred utilization of combinations of DOC fractions may add to existing observations of successions and inform ecological models at desired levels of resolution. This work might help identify whether the broad-scale metabolic preferences suggested to explain microbial succession patterns in the marine environment (30) agree with the growth preferences of heterotrophic bacteria and perhaps predict succession patterns based on heterotrophic growth. Our results suggest that these preferences are partially predictable based on phylogeny but that extensive intraclade variability requires alternative approaches to group bacteria based on their metabolic role in the oceans. For example, bacteria belonging to cluster 4 (including some *Alteromonas* and *Marinoacter* strains) would be highly competitive across a wide range of organic matter, as they grow fairly well on amino acids in addition to lipids, disaccharides, and (to some extent) amino sugars. In contrast, those belonging to cluster 1 seem to be more specifically tuned to amino acids.

While some of the associations between pathways and carbon class can be easily attributed to a direct enrichment of the corresponding metabolic utilization processes, other enrichment patterns point to processes (including nonmetabolic ones) for which the biological connection is not obvious. We hypothesize that these “cryptic” associations between carbon classes and enriched KEGG pathways carry information about environmental adaptations, suggesting a connection between the metabolic preferences of marine heterotrophs and ecologically relevant roles they play within a microbial community.

For example, we found growth on peptides to be correlated with genes whose function relates to biofilm formation, chemotaxis, and motility. Since previous studies have found these traits to be significantly enriched in microbial communities sampled from marine particles (28, 70–73), we speculate that the ability to grow robustly on peptides may be associated with a specific temporal role in the colonization of particles in marine environments. Peptide assimilation begins with the largely nonspecific extracellular cleavage of protein fragments (74, 75), enabling microbes to thrive on a wide variety of substrates. Labile organic matter, such as peptides, is turned over rapidly in seawater (76), and the ability to sense, target, and adhere to these substrates would confer a competitive advantage in the early colonization of “fresh” proteinaceous matter. Several studies have identified specific phases of algal blooms characterized by pronounced increases in peptide consumption associated with characteristic phylogenetic lineages of marine heterotrophs (30, 31, 77); similar observations have also been mirrored in mesocosm experiments (28). Our findings support the notion that a preference for peptides may be a defining feature of bacteria that thrive in specific phases of succession on marine particles or during algal blooms.

A second example is that of bacteria growing on organic acids, which we found to be strongly enriched for the ethylmalonyl-CoA pathway, possibly reflecting the importance of this pathway in the photoheterotrophic capabilities shared by many of these bacteria (60, 78). These same bacteria also display strong enrichment for vitamin B12 production (a defining characteristic of phytoplankton-heterotrophs interactions [79]), corroborating the previously suggested role of these bacteria as key partners of eukaryotic phytoplankton. These findings are also consistent with genomic and experimental evidence that photoheterotrophy is a widespread metabolic strategy among certain *Alphaproteobacteria* clades (60, 78), such as the *Rhodobacterales* from our library. Previous studies have indicated that these strains lack the genes for carbon fixation and are therefore unable to grow autotrophically but use light to supplement their energetic requirements (60, 80). This agrees with our observation that these strains did not grow in the medium lacking carbon sources. It also suggests that the ethylmalonyl-CoA pathway could play a dual role in organic acid assimilation (as supported by the enrichment mentioned above) and CO_2_ fixation, as documented in other photoheterotrophs (81). Taken together, these cryptic associations can be interpreted as correlated adaptations with a potential ecological significance, similar to recently identified linked trait clusters (49).

While the phenotypic matrix shown in Fig. 2 is generally thought in terms of its columns, representing the growth profiles of the different organisms, one can also reflect on the relevance of its rows, which display the type of communities that each DOC fraction is able to support. Notably, different carbon sources seem to support very different numbers of taxa: for example, amino acids support many different species, while amino sugars only support appreciable growth in a narrow category of bacteria. The broad utilization of amino acids by many organisms may simply reflect the fact that they may be able to directly import and use amino acids as building blocks, funneling them directly into biomass. Alternatively, it is possible that distinct sets of organisms preferentially use different individual amino acids, in a way that our current setup (where all amino acids are mixed into a single carbon fraction) would not be able to dissect. One could apply to carbon classes a categorization similar to the one used to describe microbial species as generalists or specialists. In the same way as a species that can grow on multiple nutrients is thought of as a generalist, a carbon source that can support multiple clades could be called “versatile.” A carbon fraction that only supports a small number of clades (similar to a specialist organism) could be thought of as nonversatile, or “exclusive.” In the future it will be interesting to study the distributions of versatility in different environments and their ecological implications, e.g., with consumer-resource models that use statistical ensembles of random matrices to parametrize ecological models (82).

This laboratory study only provides a glimpse of the myriad factors likely to influence heterotrophic metabolic activity in the ocean, as the complete picture of microbial growth encompasses much more than the quantity and structure of carbon sources. Prior work has indicated that the elemental stoichiometry of DOC has a strong influence on microbial community function (83). While our refactored media are designed so as to contain the same amount of carbon, we cannot exclude the possibility that the elemental ratios of the defined media affect growth beyond the effect of the specific carbon sources. A compelling next step would be to examine the effect of nutrient availability on heterotroph carbon source preference. The landscape of organic matter in the ocean is far more complex than could be represented in laboratory experiments, and biotic interactions greatly influence metabolic activity. However, some of the trends we observed have been reported by previous studies, supporting the ecological relevance of our data set (30, 84). While we do not expect the exact patterns observed in our experiments to play out in nature, our study provides a framework to continue investigating the role that heterotrophic bacteria play in carbon cycling.

## MATERIALS AND METHODS

### Selection and construction of strain library.

We assembled a library of 63 heterotrophic isolates representing major marine lineages common to both the global oligotrophic and temperate oceans (Table S1). The representative strains were determined by a comprehensive genome analysis presented in Zoccarato et al. (49), in which 473 high-quality marine microbial genomes were analyzed using a workflow aimed at detecting traits rather than the presence of individual genes. Based on the occurrence patterns of all traits, the genomes were clustered into 47 genome functional clusters (GFCs) expected to represent groups of organisms with defined ecologies and life histories. We selected representative strains from each GFC based on their availability from strain repositories or individual lab collections, applying the additional criteria that they be culturable in standard laboratory conditions (at 26°C in Marine Broth), be nonpathogenic with a BSL1 rating, and be heterotrophic. In addition, we also included five nonmarine strains. Two E. coli strains were chosen to serve as a benchmark for our experiments: E. coli Seattle 1946 (25922) is a well-studied strain commonly used for quality control in microbial phenotyping assays; the origin of E. coli strain MRE600 is not well documented but is believed to have been recovered from an environmental water sample. The remaining three strains were chosen because they were commercially available representatives of the GFCs that emerged as part of the original genomic analysis: *Cronobactor universalis* (crono) was isolated from fresh water; Shewanella denitrificans OS217 (shewden) was isolated from brackish water; and Exiguobacterium oxidotolerans T-2-2 (exig) was isolated from a water sample found at a fish processing plant. Strains were obtained from the sources listed in Table S1. Samples were streaked on marine agar, and single colonies were subsequently picked and inoculated into Marine Broth. Stocks were prepared from liquid cultures in 50% glycerol and stored at –80°C.

### Construction of refactored media.

As a first step in our analysis we compared strains according to their growth phenotypes with the goal of determining to what extent heterotrophic bacteria can utilize available marine carbon sources. We therefore took a well-known marine culture medium (Difco Marine Broth), on which all strains were experimentally determined to grow, and selected a core set of macromolecular compounds likely to comprise the undefined components of Marine Broth: yeast extract and peptone. Eight media containing different classes of carbon sources were developed, each with multiple individual components: peptides, amino acids, lipids, organic acids, disaccharides, monosaccharides, amino sugars, and acidic sugars (Table S2). A medium containing all eight classes and a negative control lacking added carbon were developed as well. In addition, nitrogen, phosphorus, and sulfur sources, as well as salts and vitamins, were added in excess to all of the refactored media. The exact quantities and components of each medium are detailed in Table S2.

Since the different media were built to approximately reproduce fractions of the Marine Broth medium, they were not uniform in their content of specific elements. In particular, 8 of the 10 refactored media have the same total mass of carbon-containing sources, but not necessarily the same number of carbon atoms (Table S8 at https://github.com/segrelab/marine_heterotrophs/). The number of carbon atoms could not be calculated for difcoMB because the exact composition of the medium is unknown; therefore, we chose to standardize the mass of total carbon source to imitate the components of difcoMB as closely as possible, although it resulted in varying amounts of carbon and nitrogen atoms across the different media (Table S8 at https://github.com/segrelab/marine_heterotrophs/ and Fig. S4A). Similarly, the number of carbon and nitrogen atoms for the medium containing peptides (and therefore the complete medium) could only be estimated because an exact molecular formula for Casamino Acids (a hydrolysate of casein) is not available. The negative control medium (HMB–) has 0 added carbon source, and HMBlips has a total of 2.2 g/L of carbon source compared to 5 g/L for the other media due to solubility issues of the lipid mixture; this combined with the much greater molecular weight of the lipid components contributes to the low number of carbon atoms in the medium (Table S8 at https://github.com/segrelab/marine_heterotrophs/ and Fig. S4A). Note that the HMBlips medium was prepared with a commercially available lipid mixture (Sigma Lipid Mixture 1), which, in addition to the lipids themselves, contains pluronic F-68 as an anti-foaming agent and Tween 80 as an emulsifier. We cannot rule out the possibility that these molecules may be metabolized by some of the bacteria, partially contributing to the growth observed on HMBlips.

### Growth assay.

Using sterile technique, strains were streaked from frozen stocks (maintained at −80°C) onto marine agar plates and placed in a 26°C incubator. Cultures were grown 72 h, then single colonies were picked and inoculated in 2 mL Marine Broth in 5-mL falcon tubes. Liquid cultures were grown with shaking (200 rpm) at 26°C and ambient light for 48 h. Negative controls without added bacteria were used to verify the absence of contamination. Starter cultures of 1.5 μL were then inoculated into each well of a 96-well plate containing 149 μL of medium, in triplicate. Only the interior wells were utilized; edge wells contained Marine Broth without added bacterial cultures to reduce evaporation and check for contamination. All combinations of strains and media were tested. Plates were wrapped with parafilm and incubated at 26°C and ambient light without shaking for 264 h. Negative controls for each medium (without added bacteria) were included in addition to edge wells. Plates were removed from the incubator individually, the lids were checked for condensation, and the optical density (600 nm) was measured in a Biotek Synergy HT plate reader (software version 3.05.11) at 26°C approximately every 24 h. Plates were shaken prior to the read at 0 h only. Results (in the form of time series of OD_600_ for each well) were downloaded from the plate reader software as excel files and analyzed using the R statistical programming language (85).

### Growth profiles.

The heterotrophs selected for the library displayed widely divergent growth dynamics, and we chose to focus on their potential to generate biomass regardless of growth rate. As described below, we used the growth curves to estimate, for each strain, a growth profile across media, capturing the maximal change in optical density achieved by that strain at any time point during the growth assay. The three technical replicates for each organism (*i*) and condition (*j*) were highly similar to each other (Fig. S1) and were averaged at each time point to produce an average growth curve OD_ij_(*t*). We next identified for all pairs *i*,*j*, the maximum value (max_t_{OD*_ij_*(*t*)}) reached by OD*_ij_*(*t*) throughout the growth time course. The normalized version of this maximum value across all strains, maxOD*_ij_* = max_t_{OD*_ij_*(*t*)}/OD*_ij_*(*t* = 0), constitutes a matrix whose row *i* represents the growth profile of organism *i* across all conditions. The matrix maxOD*_ij_* (*i* = 1, …, 63; *j* = 1, …, 10) is used for subsequent comparative analyses of heterotroph phenotypes. Following a Kruskal-Wallis test for each medium, the nonparametric *post hoc* Dunn’s test was used to determine significant growth between the test and negative control without added bacteria for each medium. The Benjamini-Hochberg correction for multiple testing was applied, and cases with *P* < 0.05 were ascribed the unchanged maxOD*_ij_* value. Conversely, maxOD*_ij_* values for strain/medium pairs that did not experience positive growth were set to 0.

Note that we chose to use optical density as a proxy for strain growth because it best captures the total biomass yield, which is most relevant for global stoichiometry in the oceans. In other words, we were interested in the ability of each organism to produce biomass on a given carbon category. Other growth metrics, such as growth rate and the time to reach the maximum OD, constitute important variables that affect meaningful ecological phenotypes, like competition for limited resources, but were not thoroughly analyzed in this study. Given that several strains display diauxic shifts and other nonlinearities during the active growth phase, these other metrics may be difficult to estimate as individual parameters and would ideally require higher time resolution. We have, however, explicitly asked whether there is any systematic relationship between maximum OD and initial growth rate. As shown in Fig. S2, the two quantities are strongly correlated (adjusted *R*^2^ = 0.71, *P* < 2.2 × 10^−16^).

### Unsupervised clustering of growth profiles.

The growth profiles (maxOD*_ij_*) of all 63 strains were clustered using a Gaussian mixture model implemented in the R mclust package (86), a contributed R package for model-based clustering, classification, and density estimation based on finite normal mixture modeling, abundantly used to analyze biological data sets (e.g., references 87–89). Model-based clustering approaches, such as gaussian mixture modeling, provide a probabilistic alternative to traditional methods, such as k-means and hierarchical clustering; the challenges of selecting the “correct” number of clusters and clustering method are resolved by statistical model selection rather than heuristic methods (90, 91). mclust provides functions for parameter estimation via the expectation maximization algorithm for normal mixture models with a variety of covariance structures and functions for simulation from these models. Initialization is performed using the partitions obtained from agglomerative hierarchical clustering. By default, mclust applies 14 models and identifies the one that best characterizes the data. mclust achieves this by computing, for each model, the Bayesian information criterion (BIC), which has been shown to work well in model-based clustering (92, 93). Specifically, BIC was used within mclust to identify the optimal covariance parameters (in our case VEI), as well as the optimal number of clusters (in our case, 7). See Fig. S6 for a comparison of BIC values obtained for all models.

10.1128/msystems.00070-22.6 FIG S6 Gaussian Mixture Model (GMM) model selection. The Bayesian information criterion (BIC) is plotted as a function of the number of clusters for 14 different parameterization approaches implemented by mclust (shown in subset, see reference 68 for details). The model with the highest BIC is identified as having the optimal parameters; in our case, VEV (Volume, Shape, Orientation = Variable, Equal, Variable) with seven clusters. Download FIG S6, TIF file, 0.6 MB. Copyright © 2022 Forchielli et al. 2022 Forchielli et al. https://creativecommons.org/licenses/by/4.0/ This content is distributed under the terms of the Creative Commons Attribution 4.0 International license.

A chi-square test of independence was performed to examine the relationship between taxonomic class and cluster number. The relationship between these variables was significant (*P* = 0.0005). The squared standardized Pearson residuals were then examined to indicate which classes of bacteria contributed significantly to the lack of fit between the observed data and null model (the taxonomic class and cluster assignment are independent); values greater than 4 were considered statistically significant at a critical alpha value of 0.05 (Table S3 at https://github.com/segrelab/marine_heterotrophs/).

### Relationship between growth profiles and phylogenetic/gene content distance.

We constructed a phylogenetic tree for all the strains in our collection using protein sequences of 206 single-copy homologous genes. The homologous genes were obtained by first conducting an all-against-all comparison of translated nucleotide sequences using BLAST (94). The BLAST pairs were then filtered for reciprocal hits and clustered using MCL (95). The sequences in each cluster were aligned with Muscle (96) and trimmed with Gblocks (97), and HMMER (98) was used to build hidden Markov models (HMMs) for each cluster. We then searched the genomes for each HMM, and the orthologs were selected based on their presence in all genomes (multiple copies were ignored). The homologous sequences for each strain were concatenated and aligned with Muscle, cleaned with Gblocks, and the final maximum-likelihood tree was inferred using FastTree (99). The cophenetic distance (*P_ab_*) between all pairs of strains was calculated using the cophenetic.phylo function of the R ape package (100).

The gene content distance (*C_ab_*) and the metabolic trait distance (KEGG module distance, *M_ab_*) for all pairs of strains were calculated as described in Zoccarato et al. (49), using the Jaccard distance. All of the genomes used in this study were reannotated with KEGG orthology (KO) identification numbers by Zoccarato et al. For Fig. 3B, we created the library pangenome by matching the genes based on their KO identifiers, ignoring duplicate genes within the same genome. We built the matrix by first taking the nonredundant union of genes for all strains in the library, and then we organized the matrix so that the rows represented strains, the columns represented the genes, and the presence or absence of genes in each strain was given a binary encoding. The gene content distance (*C_ab_*) was subsequently determined by calculating the Jaccard distance between binary vectors for all pairs of strains. The metabolic trait distance (*M_ab_*) was calculated in an identical way using binary vectors for each strain encoding the presence or absence of KEGG modules; the methodology for determining the presence of modules within each genome is detailed in reference (49) In this case, the final matrix columns are KEGG modules, which represent pathways, structural complexes (e.g., transmembrane pumps or ribosomes), functional sets (e.g., aminoacyl-tRNA synthases or nucleotide sugar biosynthesis), or signaling modules (e.g., phenotypic markers such as pathogenicity).

The distance between each pair of growth profiles (*G_ab_*) was calculated using the Euclidean distance between vectors of continuous numbers corresponding to the MaxOD values. We chose the Euclidean distance due to its suitability for microbial culture data sets with the same number of variables measured on similar scales (101–104) and because we were interested in the geometric proximity of the growth values (105). For each pair of strains, the Euclidean distance between growth profiles was plotted against the cophenetic distance, gene content distance, and KEGG module distance. Linear regression was performed using the lm function of the R base package (85).

### Correlations between growth and individual gene presence/absence.

We created the library pangenome by taking the nonredundant union of all genes across strains. The point biserial correlation coefficient (*r*) was calculated between growth on each medium and the presence (binary) of the 6,255 individual genes in the library pangenome. *P* values for *r* were adjusted using the Benjamini-Hochberg procedure and then filtered for values less than 0.05. The resulting set of *r* were considered the values for highly correlated genes. The matrix of correlation values, *r*, was subjected to principal-component analysis using the princomp function of the R base package (85).

### Pathway mapping/enrichment.

Enriched pathways were obtained by filtering the pangenome for significantly correlated genes (adjusted *P* < 0.05) and mapping the set for each medium to the KEGG database using the R clusterProfiler package (106). *P* values were calculated by the hypergeometric distribution and corrected for multiple testing using the Benjamini-Hochberg procedure. Enrichment was considered statistically significant for adjusted *P* < 0.05.

### Data availability statement.

All raw data sets and scripts used to generate the figures presented in this article are available at https://github.com/segrelab/marine_heterotrophs.

## References

[1] Overmann J, Lepleux C. 2016. Marine bacteria and archaea: diversity, adaptations, and culturability, p 21–55. *In* Stal LJ, Cretoiu MS (ed), The marine microbiome. Springer International Publishing, Cham, Switzerland.

[2] Bar-On YM, Phillips R, Milo R. 2018. The biomass distribution on Earth. Proc Natl Acad Sci USA 115:6506–6511. doi:10.1073/pnas.1711842115. 29784790 PMC6016768

[3] Pomeroy LR. 1974. The ocean’s food web, a changing paradigm. Bioscience 24:499–504. doi:10.2307/1296885.

[4] Azam F, Fenchel T, Field JG, Gray JS, Meyer-Reil LA, Thingstad F. 1983. The ecological role of water-column microbes in the sea. Mar Ecol Prog Ser 10:257–263. doi:10.3354/meps010257.

[5] Field CB, Behrenfeld MJ, Randerson JT, Falkowski P. 1998. Primary production of the biosphere: Integrating terrestrial and oceanic components. Science 281:237–240. doi:10.1126/science.281.5374.237. 9657713

[6] Moran MA, Kujawinski EB, Stubbins A, Fatland R, Aluwihare LI, Buchan A, Crump BC, Dorrestein PC, Dyhrman ST, Hess NJ, Howe B, Longnecker K, Medeiros PM, Niggemann J, Obernosterer I, Repeta DJ, Waldbauer JR. 2016. Deciphering ocean carbon in a changing world. Proc Natl Acad Sci USA 113:3143–3151. doi:10.1073/pnas.1514645113. 26951682 PMC4812754

[7] Sher D, Thompson JW, Kashtan N, Croal L, Chisholm SW. 2011. Response of Prochlorococcus ecotypes to co-culture with diverse marine bacteria. ISME J 5:1125–1132. doi:10.1038/ismej.2011.1. 21326334 PMC3146288

[8] Van Tol HM, Amin SA, Virginia Armbrust E. 2017. Ubiquitous marine bacterium inhibits diatom cell division. ISME J 11:31–42. doi:10.1038/ismej.2016.112. 27623332 PMC5315476

[9] Legendre L, Rassoulzadegan F. 1995. Plankton and nutrient dynamics in marine waters. Ophelia 41:153–172. doi:10.1080/00785236.1995.10422042.

[10] Bertrand EM, Saito MA, Rose JM, Riesselman CR, Lohan MC, Noble AE, Lee PA, DiTullio GR. 2007. Vitamin B12 and iron colimitation of phytoplankton growth in the Ross Sea. Limnol Oceanogr 52:1079–1093. doi:10.4319/lo.2007.52.3.1079.

[11] Koch F, Hattenrath-Lehmann TK, Goleski JA, Sañudo-Wilhelmy S, Fisher NS, Gobler CJ. 2012. Vitamin B1 and B12 uptake and cycling by plankton communities in coastal ecosystems. Front Microbiol 3:363. doi:10.3389/fmicb.2012.00363. 23091470 PMC3469840

[12] Tupas L, Koike I. 1991. Simultaneous uptake and regeneration of ammonium by mixed assemblages of heterotrophic marine bacteria. Mar Ecol Prog Ser 70:273–282. doi:10.3354/meps070273.

[13] Roth-Rosenberg D, Aharonovich D, Luzzatto-Knaan T, Vogts A, Zoccarato L, Eigemann F, Nago N, Grossart H-P, Voss M, Sher D. 2020. Prochlorococcus cells rely on microbial interactions rather than on chlorotic resting stages to survive long-term nutrient starvation. mBio 11:e01846-20. doi:10.1128/mBio.01846-20. 32788385 PMC7439483

[14] Azam F, Malfatti F. 2007. Microbial structuring of marine ecosystems. Nat Rev Microbiol 5:782–791. doi:10.1038/nrmicro1747. 17853906

[15] Azam F. 1998. Microbial control of oceanic carbon flux: the plot thickens. Science 280:694–696. doi:10.1126/science.280.5364.694.

[16] Dittmar T, Arnosti C. 2018. An inseparable liaison: marine microbes and nonliving organic matter, p 195–229. In Gasol JM, Kirchman DL (ed), Microbial ecology of the oceans, 3rd ed. John Wiley & Sons, Hoboken, NJ.

[17] Hertkorn N, Benner R, Frommberger M, Schmitt-Kopplin P, Witt M, Kaiser K, Kettrup A, Hedges JI. 2006. Characterization of a major refractory component of marine dissolved organic matter. Geochim Cosmochim Acta 70:2990–3010. doi:10.1016/j.gca.2006.03.021.

[18] Koch H, Dürwald A, Schweder T, Noriega-Ortega B, Vidal-Melgosa S, Hehemann J-H, Dittmar T, Freese HM, Becher D, Simon M, Wietz M. 2019. Biphasic cellular adaptations and ecological implications of Alteromonas macleodii degrading a mixture of algal polysaccharides. ISME J 13:92–103. doi:10.1038/s41396-018-0252-4. 30116038 PMC6298977

[19] Cole JJ, Findlay S, Pace ML. 1988. Bacterial production in fresh and saltwater ecosystems: a cross-system overview. Mar Ecol Prog Ser 43:1–10. doi:10.3354/meps043001.

[20] Flynn KJ, Clark DR, Xue Y. 2008. Modeling the release of dissolved organic matter by phytoplankton(1). J Phycol 44:1171–1187. doi:10.1111/j.1529-8817.2008.00562.x. 27041714

[21] Larsson U, Hagström A. 1979. Phytoplankton exudate release as an energy source for the growth of pelagic bacteria. Mar Biol 52:199–206. doi:10.1007/BF00398133.

[22] Obernosterer I, Herndl GJ. 1995. Phytoplankton extracellular release and bacterial growth: dependence on the inorganic N:P ratio. Mar Ecol Prog Ser 116:247–257. doi:10.3354/meps116247.

[23] Fuhrman JA. 1999. Marine viruses and their biogeochemical and ecological effects. Nature 399:541–548. doi:10.1038/21119. 10376593

[24] Caron DA, Hutchins DA. 2013. The effects of changing climate on microzooplankton grazing and community structure: drivers, predictions and knowledge gaps. J Plankton Res 35:235–252. doi:10.1093/plankt/fbs091.

[25] Giovannoni SJ, Stingl U. 2005. Molecular diversity and ecology of microbial plankton. Nature 437:343–348. doi:10.1038/nature04158. 16163344

[26] Salazar G, Sunagawa S. 2017. Marine microbial diversity. Curr Biol 27:R489–R494. doi:10.1016/j.cub.2017.01.017. 28586685

[27] Osterholz H, Singer G, Wemheuer B, Daniel R, Simon M, Niggemann J, Dittmar T. 2016. Deciphering associations between dissolved organic molecules and bacterial communities in a pelagic marine system. ISME J 10:1717–1730. doi:10.1038/ismej.2015.231. 26800236 PMC4918438

[28] Datta MS, Sliwerska E, Gore J, Polz MF, Cordero OX. 2016. Microbial interactions lead to rapid micro-scale successions on model marine particles. Nat Commun 7:11965. doi:10.1038/ncomms11965. 27311813 PMC4915023

[29] Pinhassi J, Havskum H, Peters F, Guadayol Ò, Malits A, Pinhassi J, Havskum H, Peters F, Malits A. 2004. Changes in bacterioplankton composition under different phytoplankton regimens. Appl Environ Microbiol 70:6753–6766. doi:10.1128/AEM.70.11.6753-6766.2004. 15528542 PMC525254

[30] Teeling H, Fuchs BM, Becher D, Klockow C, Gardebrecht A, Bennke CM, Kassabgy M, Huang S, Mann AJ, Waldmann J, Weber M, Klindworth A, Otto A, Lange J, Bernhardt J, Reinsch C, Hecker M, Peplies J, Bockelmann FD, Callies U, Gerdts G, Wichels A, Wiltshire KH, Glöckner FO, Schweder T, Amann R. 2012. Substrate-controlled succession of marine bacterioplankton populations induced by a phytoplankton bloom. Science 336:608–611. doi:10.1126/science.1218344. 22556258

[31] Buchan A, LeCleir GR, Gulvik CA, González JM. 2014. Master recyclers: features and functions of bacteria associated with phytoplankton blooms. Nat Rev Microbiol 12:686–698. doi:10.1038/nrmicro3326. 25134618

[32] Amin SA, Hmelo LR, Van Tol HM, Durham BP, Carlson LT, Heal KR, Morales RL, Berthiaume CT, Parker MS, Djunaedi B, Ingalls AE, Parsek MR, Moran MA, Armbrust EV. 2015. Interaction and signalling between a cosmopolitan phytoplankton and associated bacteria. Nature 522:98–101. doi:10.1038/nature14488. 26017307

[33] Durham BP, Boysen AK, Carlson LT, Groussman RD, Heal KR, Cain KR, Morales RL, Coesel SN, Morris RM, Ingalls AE, Armbrust EV. 2019. Sulfonate-based networks between eukaryotic phytoplankton and heterotrophic bacteria in the surface ocean. Nat Microbiol 4:1706–1715. doi:10.1038/s41564-019-0507-5. 31332382

[34] Zubkov MV, Tarran GA, Mary I, Fuchs BM. 2007. Differential microbial uptake of dissolved amino acids and amino sugars in surface waters of the Atlantic Ocean. J Plankton Res 30:211–220. doi:10.1093/plankt/fbm091.

[35] Neufeld JD, Boden R, Moussard H, Schäfer H, Murrell JC. 2008. Substrate-specific clades of active marine methylotrophs associated with a phytoplankton bloom in a temperate coastal environment. Appl Environ Microbiol 74:7321–7328. doi:10.1128/AEM.01266-08. 18849453 PMC2592898

[36] Ferrer-González FX, Widner B, Holderman NR, Glushka J, Edison AS, Kujawinski EB, Moran MA. 2021. Resource partitioning of phytoplankton metabolites that support bacterial heterotrophy. ISME J 15:762–773. doi:10.1038/s41396-020-00811-y. 33097854 PMC8027193

[37] Weitz JS, Stock CA, Wilhelm SW, Bourouiba L, Coleman ML, Buchan A, Follows MJ, Fuhrman JA, Jover LF, Lennon JT, Middelboe M, Sonderegger DL, Suttle CA, Taylor BP, Frede Thingstad T, Wilson WH, Eric Wommack K. 2015. A multitrophic model to quantify the effects of marine viruses on microbial food webs and ecosystem processes. ISME J 9:1352–1364. doi:10.1038/ismej.2014.220. 25635642 PMC4438322

[38] Reed DC, Algar CK, Huber JA, Dick GJ. 2014. Gene-centric approach to integrating environmental genomics and biogeochemical models. Proc Natl Acad Sci USA 111:1879–1884. doi:10.1073/pnas.1313713111. 24449851 PMC3918765

[39] Burga A, Lehner B. 2012. Beyond genotype to phenotype: why the phenotype of an individual cannot always be predicted from their genome sequence and the environment that they experience. FEBS J 279:3765–3775. doi:10.1111/j.1742-4658.2012.08810.x. 22934970

[40] Lehner B. 2013. Genotype to phenotype: lessons from model organisms for human genetics. Nat Rev Genet 14:168–178. doi:10.1038/nrg3404. 23358379

[41] Doyle RM, O’Sullivan DM, Aller SD, Bruchmann S, Clark T, Coello Pelegrin A, Cormican M, Diez Benavente E, Ellington MJ, McGrath E, Motro Y, Phuong Thuy Nguyen T, Phelan J, Shaw LP, Stabler RA, van Belkum A, van Dorp L, Woodford N, Moran-Gilad J, Huggett JF, Harris KA. 2020. Discordant bioinformatic predictions of antimicrobial resistance from whole-genome sequencing data of bacterial isolates: an inter-laboratory study. Microb Genom 6:e000335. doi:10.1099/mgen.0.000335. PMC7067211 32048983

[42] Mallick H, Franzosa EA, Mclver LJ, Banerjee S, Sirota-Madi A, Kostic AD, Clish CB, Vlamakis H, Xavier RJ, Huttenhower C. 2019. Predictive metabolomic profiling of microbial communities using amplicon or metagenomic sequences. Nat Commun 10:3136. doi:10.1038/s41467-019-10927-1. 31316056 PMC6637180

[43] Choi J, Yang F, Stepanauskas R, Cardenas E, Garoutte A, Williams R, Flater J, Tiedje JM, Hofmockel KS, Gelder B, Howe A. 2017. Strategies to improve reference databases for soil microbiomes. ISME J 11:829–834. doi:10.1038/ismej.2016.168. 27935589 PMC5364351

[44] Tramontano M, Andrejev S, Pruteanu M, Klünemann M, Kuhn M, Galardini M, Jouhten P, Zelezniak A, Zeller G, Bork P, Typas A, Patil KR. 2018. Nutritional preferences of human gut bacteria reveal their metabolic idiosyncrasies. Nat Microbiol 3:514–522. doi:10.1038/s41564-018-0123-9. 29556107

[45] Ernebjerg M, Kishony R. 2012. Distinct growth strategies of soil bacteria as revealed by large-scale colony tracking. Appl Environ Microbiol 78:1345–1352. doi:10.1128/AEM.06585-11. 22194284 PMC3294487

[46] Wiegand S, Jogler M, Boedeker C, Pinto D, Vollmers J, Rivas-Marín E, Kohn T, Peeters SH, Heuer A, Rast P, Oberbeckmann S, Bunk B, Jeske O, Meyerdierks A, Storesund JE, Kallscheuer N, Lücker S, Lage OM, Pohl T, Merkel BJ, Hornburger P, Müller RW, Brümmer F, Labrenz M, Spormann AM, Op den Camp HJM, Overmann J, Amann R, Jetten MSM, Mascher T, Medema MH, Devos DP, Kaster AK, Øvreås L, Rohde M, Galperin MY, Jogler C. 2020. Cultivation and functional characterization of 79 planctomycetes uncovers their unique biology. Nat Microbiol 5:126–140. doi:10.1038/s41564-019-0588-1. 31740763 PMC7286433

[47] O’Brien EJ, Monk JM, Palsson BO. 2015. Using genome-scale models to predict biological capabilities. Cell 161:971–987. doi:10.1016/j.cell.2015.05.019. 26000478 PMC4451052

[48] Klitgord N, Segrè D. 2010. Environments that induce synthetic microbial ecosystems. PLoS Comput Biol 6:e1001002. doi:10.1371/journal.pcbi.1001002. 21124952 PMC2987903

[49] Zoccarato L, Sher D, Miki T, Segrè D, Grossart H-P. 2022. A comparative whole-genome approach identifies bacterial traits for marine microbial interactions. Commun Biol 5:276. doi:10.1038/s42003-022-03184-4. 35347228 PMC8960797

[50] Kujawinski EB. 2011. The impact of microbial metabolism on marine dissolved organic matter. Annu Rev Mar Sci 3:567–599. doi:10.1146/annurev-marine-120308-081003. 21329217

[51] Jiao N, Herndl GJ, Hansell DA, Benner R, Kattner G, Wilhelm SW, Kirchman DL, Weinbauer MG, Luo T, Chen F, Azam F. 2010. Microbial production of recalcitrant dissolved organic matter: long-term carbon storage in the global ocean. Nat Rev Microbiol 8:593–599. doi:10.1038/nrmicro2386. 20601964

[52] Teske A, Durbin A, Ziervogel K, Cox C, Arnosti C. 2011. Microbial community composition and function in permanently cold seawater and sediments from an arctic fjord of svalbard. Appl Environ Microbiol 77:2008–2018. doi:10.1128/AEM.01507-10. 21257812 PMC3067321

[53] Beier S, Bertilsson S. 2013. Bacterial chitin degradation-mechanisms and ecophysiological strategies. Front Microbiol 4:149. doi:10.3389/fmicb.2013.00149. 23785358 PMC3682446

[54] Kirchman D, Hodson R. 1984. Inhibition by peptides of amino acid uptake by bacterial populations in natural waters: implications for the regulation of amino acid transport and incorporation. Appl Environ Microbiol 47:624–631. doi:10.1128/aem.47.4.624-631.1984. 16346504 PMC239738

[55] Kirchman DL. 1990. Limitation of bacterial growth by dissolved organic matter in the subarctic Pacific. Mar Ecol Prog Ser 62:47–54. doi:10.3354/meps062047.

[56] Payne WJ, Wiebe WJ. 1978. Growth yield and efficiency in chemosynthetic microorganisms. Annu Rev Microbiol 32:155–183. doi:10.1146/annurev.mi.32.100178.001103. 30389

[57] Goodhue CT, Snell EE. 1966. The bacterial degradation of pantothenic acid. I. Over-all nature of the reaction. Biochemistry 5:393–398. doi:10.1021/bi00866a001. 5940927

[58] Rontani JF, Bonin PC, Volkman JK. 1999. Production of wax esters during aerobic growth of marine bacteria on isoprenoid compounds. Appl Environ Microbiol 65:221–230. doi:10.1128/AEM.65.1.221-230.1999. 9872783 PMC91006

[59] Alvarez HM, Pucci OH, Steinbüchel A. 1997. Lipid storage compounds in marine bacteria. Appl Microbiol Biotechnol 47:132–139. doi:10.1007/s002530050901.

[60] Moran MA, Miller WL. 2007. Resourceful heterotrophs make the most of light in the coastal ocean. Nat Rev Microbiol 5:792–800. doi:10.1038/nrmicro1746. 17828280

[61] Luli GW, Strohl WR. 1990. Comparison of growth, acetate production, and acetate inhibition of Escherichia coli strains in batch and fed-batch fermentations. Appl Environ Microbiol 56:1004–1011. doi:10.1128/aem.56.4.1004-1011.1990. 2187400 PMC184335

[62] Pinhal S, Ropers D, Geiselmann J, de Jong H. 2019. Acetate metabolism and the inhibition of bacterial growth by acetate. J Bacteriol 201 doi:10.1128/JB.00147-19. PMC6560135 30988035

[63] Carpenter CE, Broadbent JR. 2009. External concentration of organic acid anions and pH: key independent variables for studying how organic acids inhibit growth of bacteria in mildly acidic foods. J Food Sci 74:R12–5. doi:10.1111/j.1750-3841.2008.00994.x. 19200113

[64] Roe AJ, O’Byrne C, McLaggan D, Booth IR. 2002. Inhibition of Escherichia coli growth by acetic acid: a problem with methionine biosynthesis and homocysteine toxicity. Microbiology (Reading) 148:2215–2222. doi:10.1099/00221287-148-7-2215. 12101308

[65] Barbara GM, Mitchell JG. 2003. Marine bacterial organisation around point-like sources of amino acids. FEMS Microbiol Ecol 43:99–109. doi:10.1111/j.1574-6941.2003.tb01049.x. 19719700

[66] Peyraud R, Kiefer P, Christen P, Massou S, Portais J-C, Vorholt JA. 2009. Demonstration of the ethylmalonyl-CoA pathway by using 13C metabolomics. Proc Natl Acad Sci USA 106:4846–4851. doi:10.1073/pnas.0810932106. 19261854 PMC2660752

[67] Kornberg HL, Beevers H. 1957. The glyoxylate cycle as a stage in the conversion of fat to carbohydrate in castor beans. Biochim Biophys Acta 26:531–537. doi:10.1016/0006-3002(57)90101-4. 13499412

[68] Kornberg HL, Krebs HA. 1957. Synthesis of cell constituents from C2-units by a modified tricarboxylic acid cycle. Nature 179:988–991. doi:10.1038/179988a0. 13430766

[69] Ensign SA. 2006. Revisiting the glyoxylate cycle: alternate pathways for microbial acetate assimilation. Mol Microbiol 61:274–276. doi:10.1111/j.1365-2958.2006.05247.x. 16856935

[70] Fontanez KM, Eppley JM, Samo TJ, Karl DM, DeLong EF. 2015. Microbial community structure and function on sinking particles in the North Pacific Subtropical Gyre. Front Microbiol 6:469. doi:10.3389/fmicb.2015.00469. 26042105 PMC4436931

[71] Stocker R, Seymour JR. 2012. Ecology and physics of bacterial chemotaxis in the ocean. Microbiol Mol Biol Rev 76:792–812. doi:10.1128/MMBR.00029-12. 23204367 PMC3510523

[72] Keegstra JM, Carrara F, Stocker R. The ecological roles of bacterial chemotaxis. Nat Rev Microbiol, in press. doi:10.1038/s41579-022-00709-w. 35292761

[73] Dang H, Lovell CR. 2016. Microbial surface colonization and biofilm development in marine environments. Microbiol Mol Biol Rev 80:91–138. doi:10.1128/MMBR.00037-15. 26700108 PMC4711185

[74] Odagami T, Morita J, Takama K, Suzuki S. 1994. Substrate specificities of extracellular proteases produced by marine putrefactive bacteria, Shewanella putrefaciens and Alteromonas haloplanktis. Lett Appl Microbiol 18:50–52. doi:10.1111/j.1472-765X.1994.tb00799.x.

[75] Obayashi Y, Suzuki S. 2005. Proteolytic enzymes in coastal surface seawater: significant activity of endopeptidases and exopeptidases. Limnol Oceanogr 50:722–726. doi:10.4319/lo.2005.50.2.0722.

[76] Nagata T. 2008. Organic matter–bacteria interactions in seawater, p 207–241. *In* Microbial ecology of the oceans. John Wiley & Sons, Inc., Hoboken, NJ.

[77] Needham DM, Fuhrman JA. 2016. Pronounced daily succession of phytoplankton, archaea and bacteria following a spring bloom. Nat Microbiol 1:16005. doi:10.1038/nmicrobiol.2016.5. 27572439

[78] Newton RJ, Griffin LE, Bowles KM, Meile C, Gifford S, Givens CE, Howard EC, King E, Oakley CA, Reisch CR, Rinta-Kanto JM, Sharma S, Sun S, Varaljay V, Vila-Costa M, Westrich JR, Moran MA. 2010. Genome characteristics of a generalist marine bacterial lineage. ISME J 4:784–798. doi:10.1038/ismej.2009.150. 20072162

[79] Geng H, Belas R. 2010. Molecular mechanisms underlying roseobacter-phytoplankton symbioses. Curr Opin Biotechnol 21:332–338. doi:10.1016/j.copbio.2010.03.013. 20399092

[80] Stoecker DK, Hansen PJ, Caron DA, Mitra A. 2017. Mixotrophy in the marine plankton. Annu Rev Mar Sci 9:311–335. doi:10.1146/annurev-marine-010816-060617. 27483121

[81] Bill N, Tomasch J, Riemer A, Müller K, Kleist S, Schmidt-Hohagen K, Wagner-Döbler I, Schomburg D. 2017. Fixation of CO2 using the ethylmalonyl-CoA pathway in the photoheterotrophic marine bacterium Dinoroseobacter shibae. Environ Microbiol 19:2645–2660. doi:10.1111/1462-2920.13746. 28371065

[82] Marsland R, III, Cui W, Mehta P. 2020. A minimal model for microbial biodiversity can reproduce experimentally observed ecological patterns. Sci Rep 10:3308. doi:10.1038/s41598-020-60130-2. 32094388 PMC7039880

[83] D’Andrilli J, Junker JR, Smith HJ, Scholl EA, Foreman CM. 2019. DOM composition alters ecosystem function during microbial processing of isolated sources. Biogeochemistry 142:281–298. doi:10.1007/s10533-018-00534-5.

[84] Bryson S, Li Z, Chavez F, Weber PK, Pett-Ridge J, Hettich RL, Pan C, Mayali X, Mueller RS. 2017. Phylogenetically conserved resource partitioning in the coastal microbial loop. ISME J 11:2781–2792. doi:10.1038/ismej.2017.128. 28800138 PMC5702734

[85] R Core Team. 2020. R: A language and environment for statistical computing. R Foundation for Statistical Computing, Vienna, Austria. https://www.R-project.org/.

[86] Scrucca L, Fop M, Murphy TB, Raftery AE. 2016. mclust 5: clustering, classification and density estimation using Gaussian finite mixture models. R J 8:289–317. doi:10.32614/RJ-2016-021. 27818791 PMC5096736

[87] Weissman JL, Hou S, Fuhrman JA. 2021. Estimating maximal microbial growth rates from cultures, metagenomes, and single cells via codon usage patterns. Proc Natl Acad Sci USA 118:e2016810118. doi:10.1073/pnas.2016810118. 33723043 PMC8000110

[88] Mehtonen J, Pölönen P, Häyrynen S, Dufva O, Lin J, Liuksiala T, Granberg K, Lohi O, Hautamäki V, Nykter M, Heinäniemi M. 2019. Data-driven characterization of molecular phenotypes across heterogeneous sample collections. Nucleic Acids Res 47:e76. doi:10.1093/nar/gkz281. 31329928 PMC6648337

[89] Enke TN, Datta MS, Schwartzman J, Cermak N, Schmitz D, Barrere J, Pascual-García A, Cordero OX. 2019. Modular assembly of polysaccharide-degrading marine microbial communities. Curr Biol 29:1528–1535.e6. doi:10.1016/j.cub.2019.03.047. 31031118

[90] Yeung KY, Fraley C, Murua A, Raftery AE, Ruzzo WL. 2001. Model-based clustering and data transformations for gene expression data. Bioinformatics 17:977–987. doi:10.1093/bioinformatics/17.10.977. 11673243

[91] Fraley C, Raftery AE. 2002. Model-based clustering, discriminant analysis, and density estimation. J Am Stat Assoc 97:611–631. doi:10.1198/016214502760047131.

[92] Dasgupta A, Raftery AE. 1998. Detecting features in spatial point processes with clutter via model-based clustering. J Am Stat Assoc 93:294–302. doi:10.1080/01621459.1998.10474110.

[93] Fraley C, Raftery AE. 1998. How many clusters? which clustering method? Answers via model-based cluster analysis. Comput J 41:578–588. doi:10.1093/comjnl/41.8.578.

[94] Boratyn GM, Camacho C, Cooper PS, Coulouris G, Fong A, Ma N, Madden TL, Matten WT, McGinnis SD, Merezhuk Y, Raytselis Y, Sayers EW, Tao T, Ye J, Zaretskaya I. 2013. BLAST: a more efficient report with usability improvements. Nucleic Acids Res 41:W29–33. doi:10.1093/nar/gkt282. 23609542 PMC3692093

[95] Enright AJ, Van Dongen S, Ouzounis CA. 2002. An efficient algorithm for large-scale detection of protein families. Nucleic Acids Res 30:1575–1584. doi:10.1093/nar/30.7.1575. 11917018 PMC101833

[96] Edgar RC. 2004. MUSCLE: multiple sequence alignment with high accuracy and high throughput. Nucleic Acids Res 32:1792–1797. doi:10.1093/nar/gkh340. 15034147 PMC390337

[97] Talavera G, Castresana J. 2007. Improvement of phylogenies after removing divergent and ambiguously aligned blocks from protein sequence alignments. Syst Biol 56:564–577. doi:10.1080/10635150701472164. 17654362

[98] Eddy SR. 1998. Profile hidden Markov models. Bioinformatics 14:755–763. doi:10.1093/bioinformatics/14.9.755. 9918945

[99] Price MN, Dehal PS, Arkin AP. 2010. FastTree 2–approximately maximum-likelihood trees for large alignments. PLoS One 5:e9490. doi:10.1371/journal.pone.0009490. 20224823 PMC2835736

[100] Paradis E, Schliep K. 2019. ape 5.0: an environment for modern phylogenetics and evolutionary analyses in R. Bioinformatics 35:526–528. doi:10.1093/bioinformatics/bty633. 30016406

[101] Legendre P, Legendre L. 2012. Numerical ecology. Elsevier, Amsterdam, The Netherlands.

[102] Queen JP, Quinn GP, Keough MJ. 2002. Experimental design and data analysis for biologists. Cambridge University Press, Cambridge, UK.

[103] Engelbrektson AL, Korzenik JR, Sanders ME, Clement BG, Leyer G, Klaenhammer TR, Kitts CL. 2006. Analysis of treatment effects on the microbial ecology of the human intestine. FEMS Microbiol Ecol 57:239–250. doi:10.1111/j.1574-6941.2006.00112.x. 16867142

[104] Rees GN, Baldwin DS, Watson GO, Perryman S, Nielsen DL. 2004. Ordination and significance testing of microbial community composition derived from terminal restriction fragment length polymorphisms: application of multivariate statistics. Antonie Van Leeuwenhoek 86:339–347. doi:10.1007/s10482-004-0498-x. 15702386

[105] Zapala MA, Schork NJ. 2006. Multivariate regression analysis of distance matrices for testing associations between gene expression patterns and related variables. Proc Natl Acad Sci USA 103:19430–19435. doi:10.1073/pnas.0609333103. 17146048 PMC1748243

[106] Wu T, Hu E, Xu S, Chen M, Guo P, Dai Z, Feng T, Zhou L, Tang W, Zhan L, Fu X, Liu S, Bo X, Yu G. 2021. clusterProfiler 4.0: a universal enrichment tool for interpreting omics data. Innovation (Camb) 2:100141. doi:10.1016/j.xinn.2021.100141. 34557778 PMC8454663

