# Supplementary figures and images for: Metabolic Phenotyping of Marine Heterotrophs on Refactored Media Reveals Diverse Metabolic Adaptations and Lifestyle Strategies
Source: mSystems. 2022 Jul 20;7(4):e00070-22. doi: 10.1128/msystems.00070-22 (PMC9426600; doi:10.1128/msystems.00070-22)

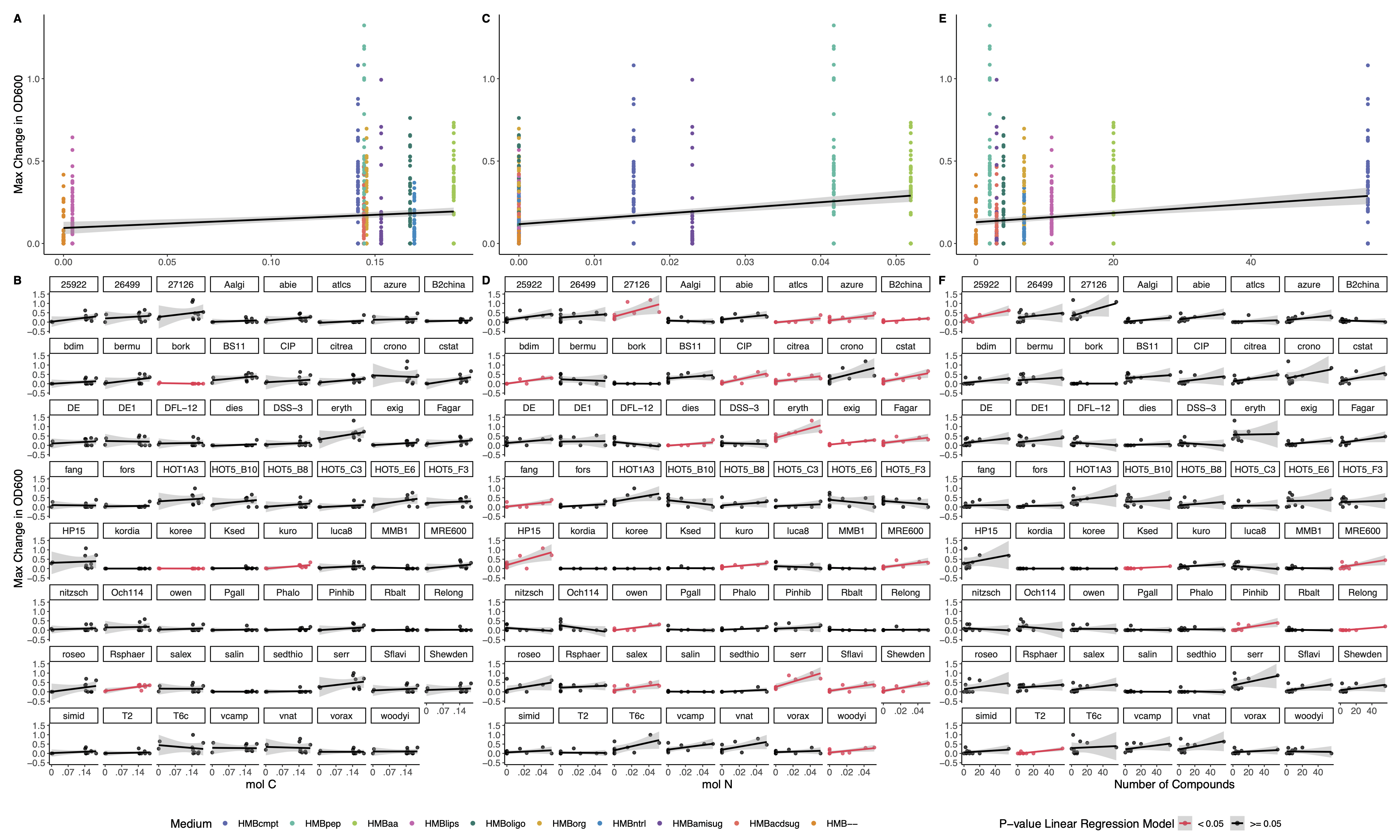

Supplement: FIG S4 [file msystems.00070-22-s0004.tif]

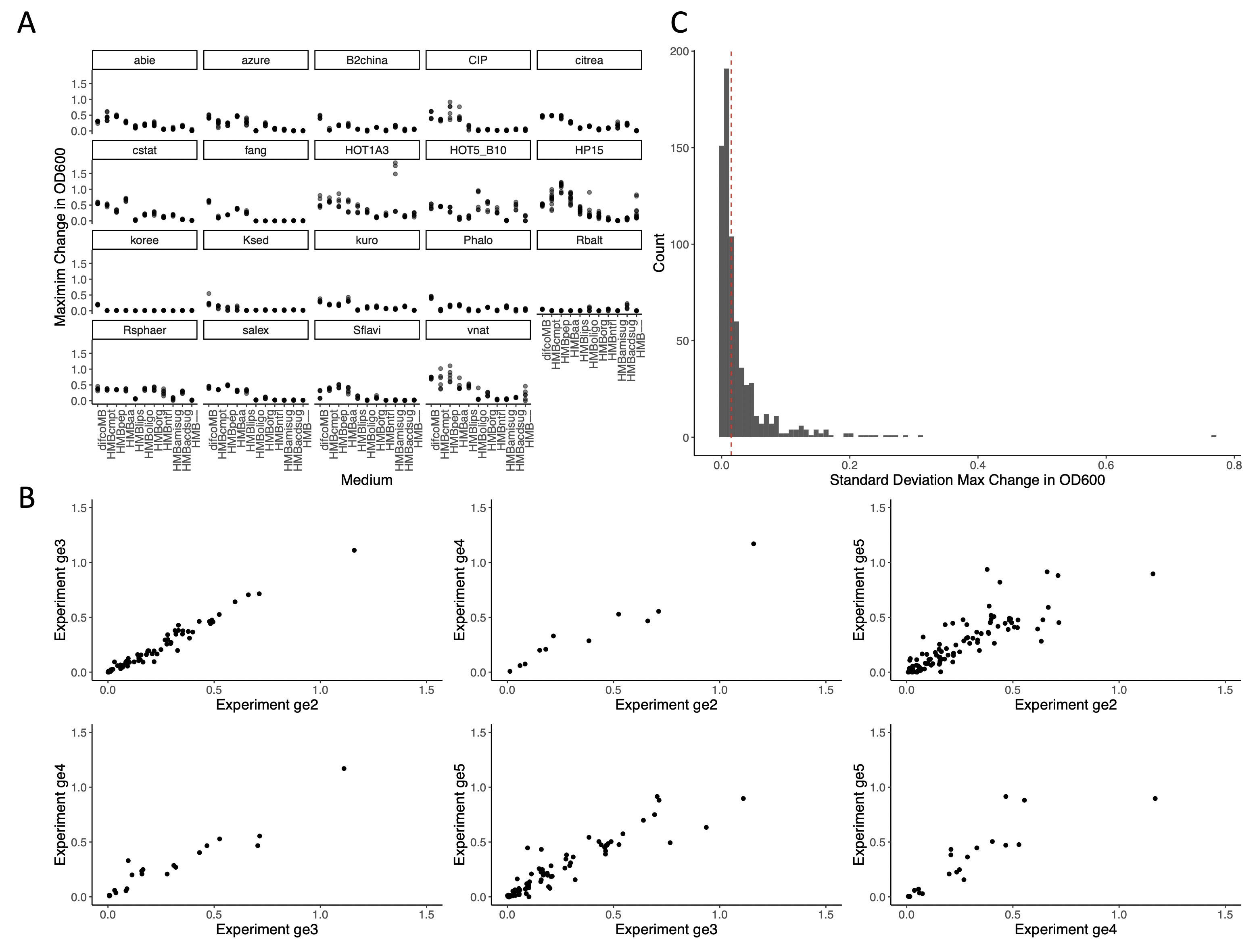

Supplement: FIG S1 [file msystems.00070-22-s0001.tif]

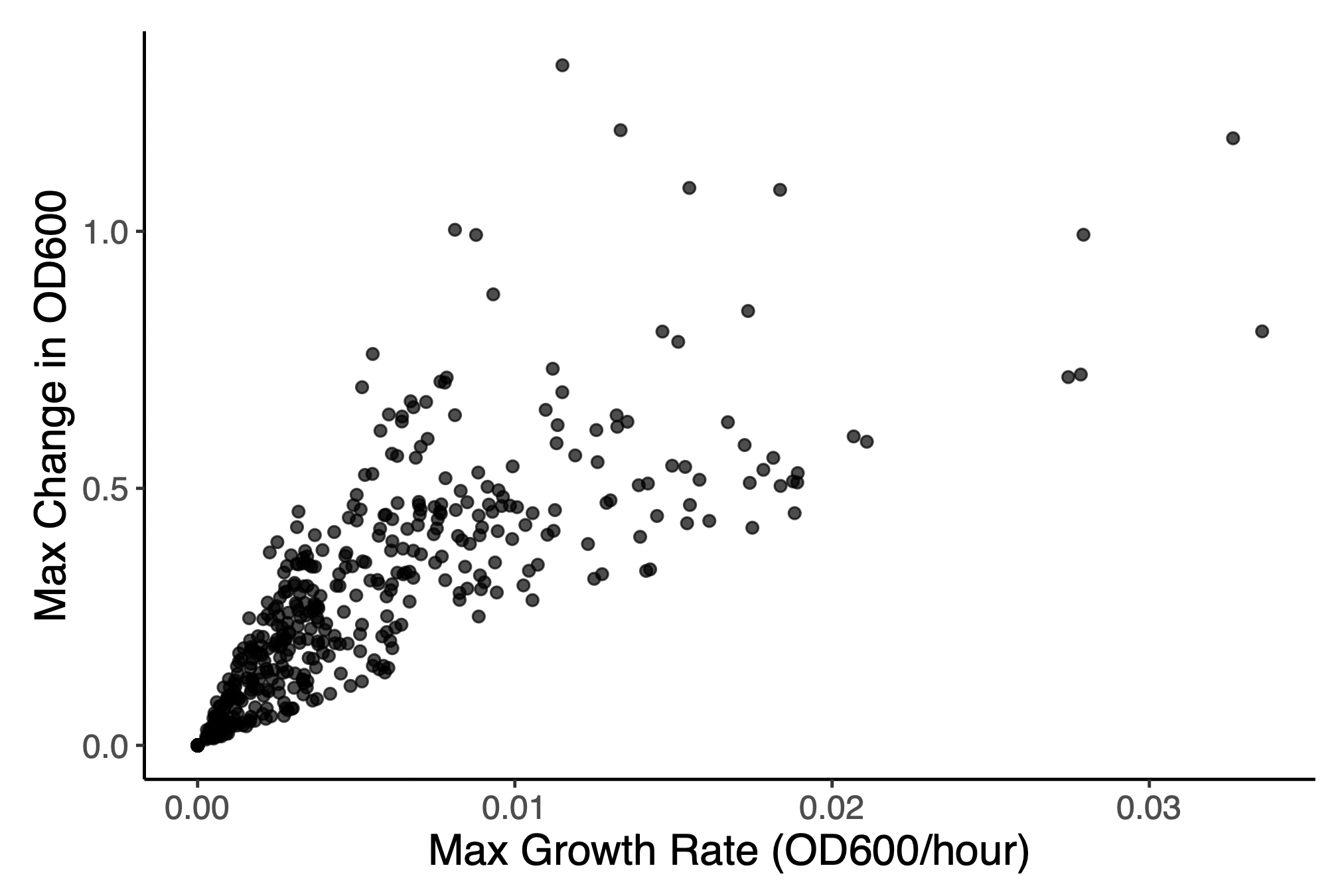

Supplement: FIG S2 [file msystems.00070-22-s0002.tif]

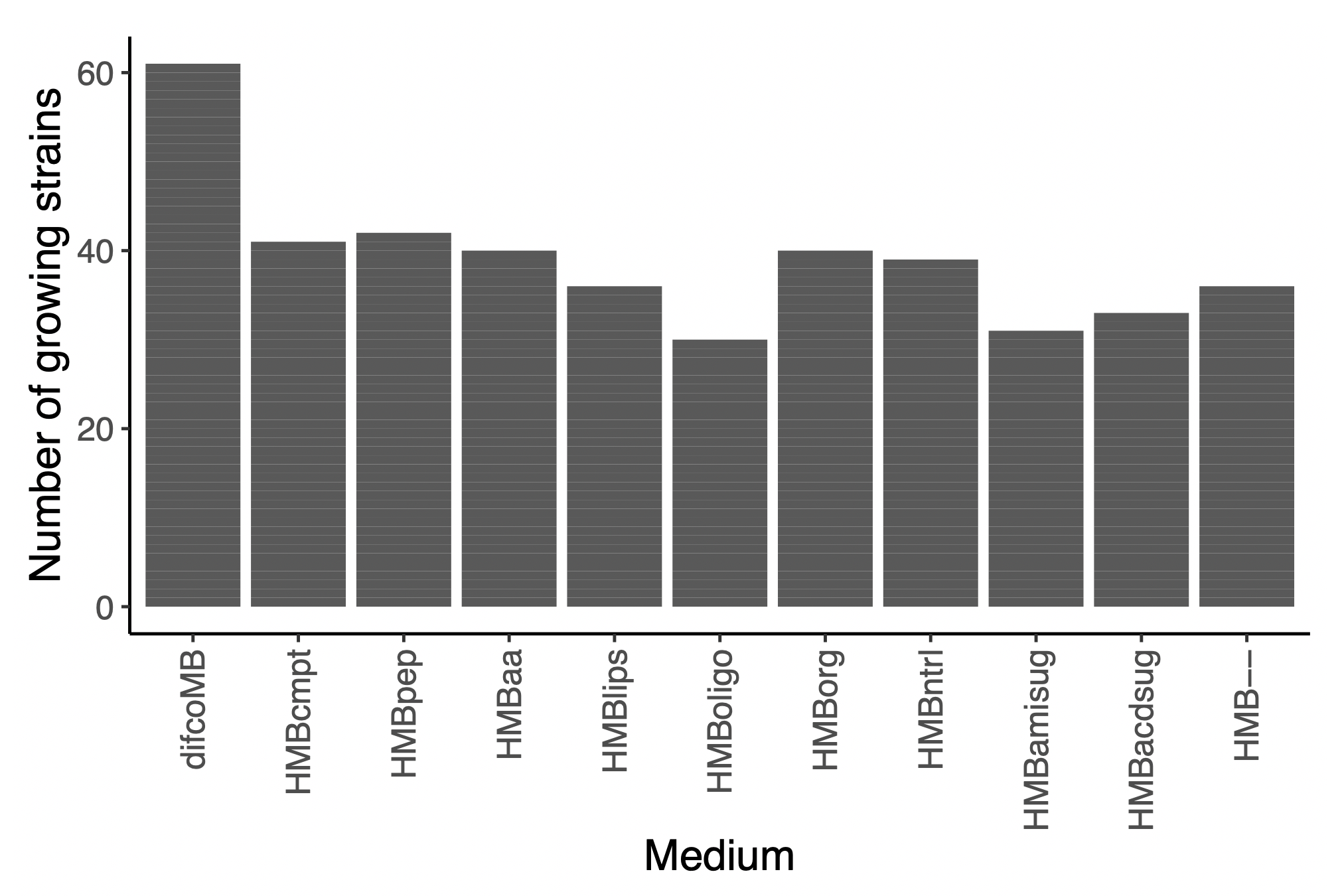

Supplement: FIG S3 [file msystems.00070-22-s0003.tif]

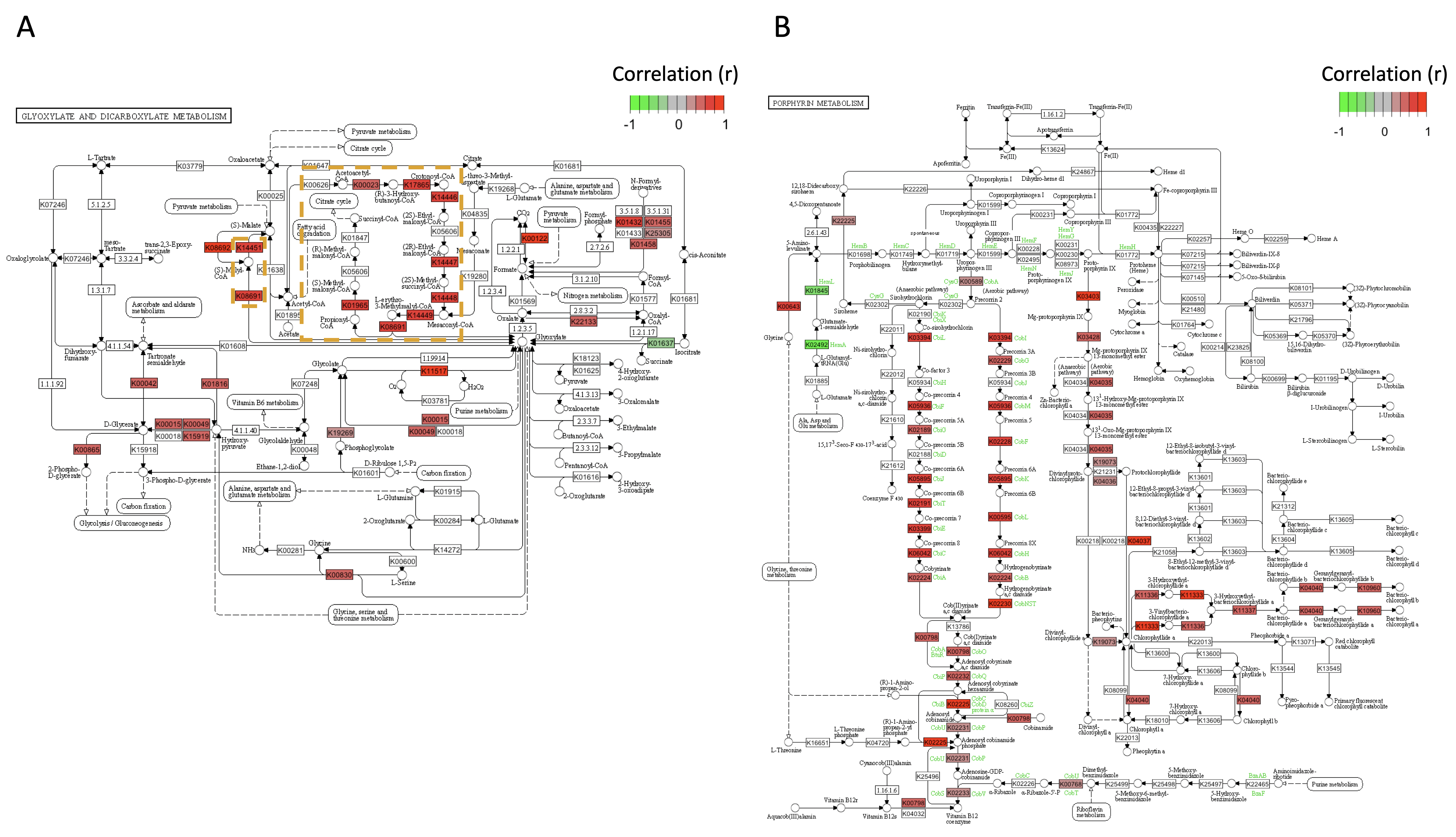

Supplement: FIG S5 [file msystems.00070-22-s0005.tif]

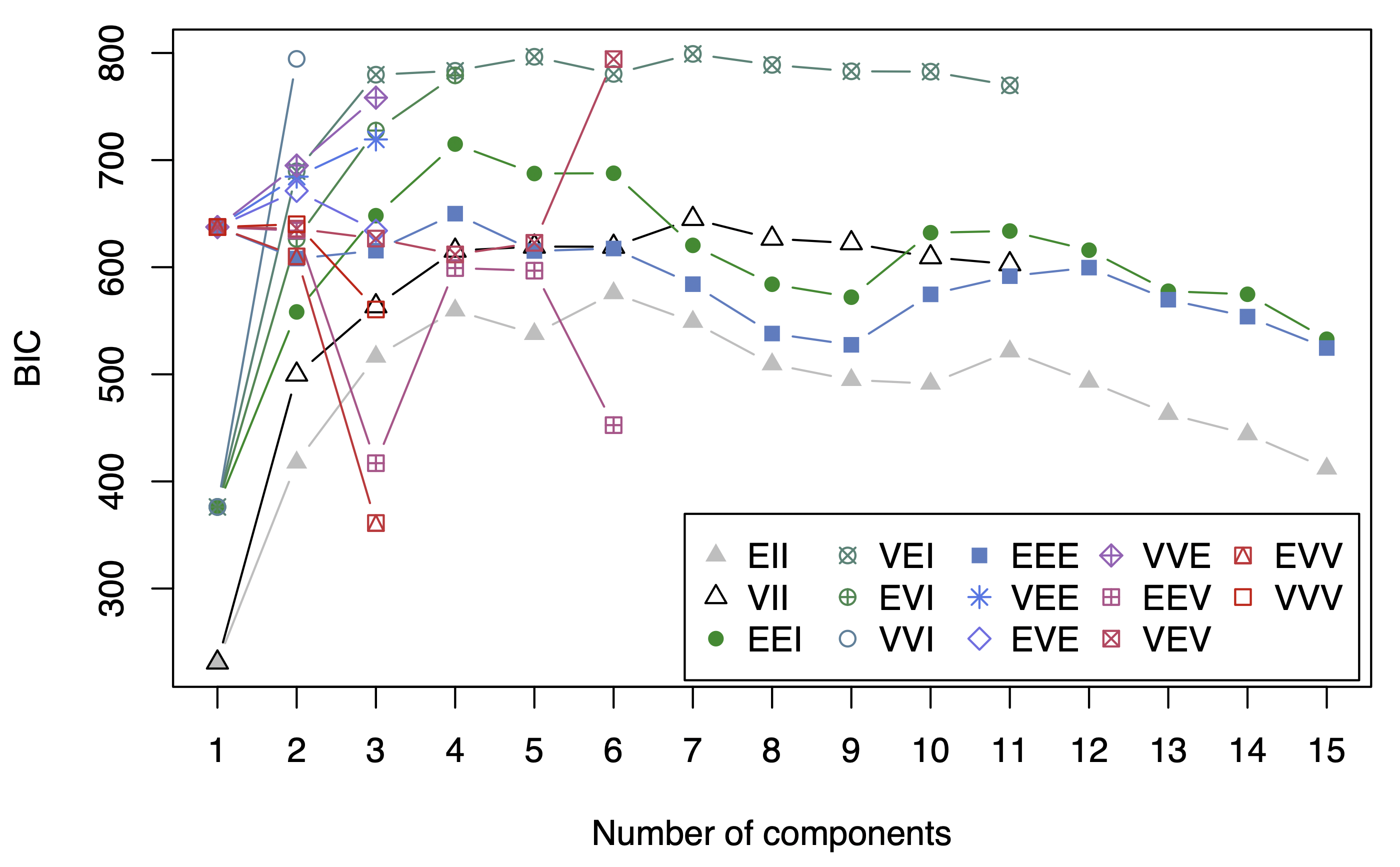

Supplement: FIG S6 [file msystems.00070-22-s0006.tif]
