# Supplementary material for: Metabolic Phenotyping of Marine Heterotrophs on Refactored Media Reveals Diverse Metabolic Adaptations and Lifestyle Strategies
Source: mSystems. 2022 Jul 20;7(4):e00070-22. doi: 10.1128/msystems.00070-22 (PMC9426600; doi:10.1128/msystems.00070-22)
Supplement: TEXT S1 [file msystems.00070-22-s0009.docx]

## **Supplementary Text**

**Impact of media stoichiometry on growth**

We performed a linear regression analysis to examine whether bacterial growth could be explained exclusively by the number of carbon or nitrogen atoms available in the media (Figure S4A, S4C). Overall, there is a statistically significant but weak relationship between the number of carbon and nitrogen atoms and change in OD600 (Adjusted R-squared = 0.022 and p-value = 9.5x10^-5^ for C atoms; Adjusted R-squared = 0.079 and p-value = 3.5x10^-13^ for N atoms), which suggests that the number of carbon and nitrogen atoms present in the media accounts for only a minor portion of the observed change in biomass. We repeated the analysis for individual strains and found that there is a statistically significant relationship for only a subset of strains and that the number of affected strains and the magnitude of the influence were both greater for nitrogen compared to carbon (Figure S4B, S4D, Supplementary Table S9 at [https://github.com/segrelab/marine_heterotrophs/]). Across all strains, the average change in OD600 per added mol was 6.97 OD600/mol for N and 0.586 OD600/mol for C. For two of the strains (koree and bork), the relationship between C atoms and growth was negative.

In addition to varying quantities of carbon and nitrogen atoms, the media also differ in the total number of added components. The number of added compounds could not be determined for difcoMB, and could only be estimated for HMBpep (see above). We performed a linear regression analysis to examine whether bacterial growth could be explained exclusively by the number of carbon sources added to the media (Figure S4E). The results were very similar to those for the abundance of carbon and nitrogen atoms: there is a statistically significant but relatively weak linear relationship between the number of components and change in OD600 (Adjusted R-squared = 0.041 p-value = 1.61x10^-7^). When examine individually, 6 of the 63 strains displayed significant relationships between the number of compounds and growth (Figure S4F), but the magnitude of this effect is much smaller compared to the effect of C and N atoms: the average change in OD600 for every increase in number of compounds is only 0.0053 (Supplementary Table S9 at [https://github.com/segrelab/marine_heterotrophs/]). It is important to note that media were not designed to explicitly examine the influence of media stoichiometry on growth; an experiment designed for this purpose may reveal a biological relationship that cannot be seen here. Taken together, whether we look at the strains collectively and individually, our regression analysis suggests that media stoichiometry may play a role in the observed bacterial growth, but its influence is likely limited in magnitude and to a subset of strains only relative to the influence of inter-strain variability; it is clear from our data that there are factors in addition to the possible contributions of carbon and nitrogen abundance that exert a powerful influence on biomass change.
